# Imaging of bacterial multicellular behaviour in biofilms in liquid by atmospheric scanning electron microscopy

**DOI:** 10.1038/srep25889

**Published:** 2016-05-16

**Authors:** Shinya Sugimoto, Ken-ichi Okuda, Reina Miyakawa, Mari Sato, Ken-ichi Arita-Morioka, Akio Chiba, Kunitoshi Yamanaka, Teru Ogura, Yoshimitsu Mizunoe, Chikara Sato

**Affiliations:** 1 Department of Bacteriology, The Jikei University School of Medicine, 3-25-8 Nishi-Shimbashi, Minato-ku, Tokyo 105-8461, Japan; 2 Jikei Center for Biofilm Science and Technology, The Jikei University School of Medicine, 3-25-8 Nishi-Shimbashi, Minato-ku, Tokyo 105-8461, Japan; 3 Biomedical Research Institute, National Institute of Advanced Industrial Science and Technology (AIST), Higashi 1-1-1, Tsukuba, Ibaraki 305-8566, Japan; 4 Department of Molecular Cell Biology, Institute of Molecular Embryology and Genetics, Kumamoto University, Chuo-Ku, Kumamoto, 860-0811, Japan

## Abstract

Biofilms are complex communities of microbes that attach to biotic or abiotic surfaces causing chronic infectious diseases. Within a biofilm, microbes are embedded in a self-produced soft extracellular matrix (ECM), which protects them from the host immune system and antibiotics. The nanoscale visualisation of delicate biofilms in liquid is challenging. Here, we develop atmospheric scanning electron microscopy (ASEM) to visualise Gram-positive and -negative bacterial biofilms immersed in aqueous solution. Biofilms cultured on electron-transparent film were directly imaged from below using the inverted SEM, allowing the formation of the region near the substrate to be studied at high resolution. We visualised intercellular nanostructures and the exocytosis of membrane vesicles, and linked the latter to the trafficking of cargos, including cytoplasmic proteins and the toxins hemolysin and coagulase. A thick dendritic nanotube network was observed between microbes, suggesting multicellular communication in biofilms. A universal immuno-labelling system was developed for biofilms and tested on various examples, including *S. aureus* biofilms. In the ECM, fine DNA and protein networks were visualised and the precise distribution of protein complexes was determined (*e.g.*, straight curli, flagella, and excreted cytoplasmic molecular chaperones). Our observations provide structural insights into bacteria-substratum interactions, biofilm development and the internal microbe community.

Biofilms are highly organized microbial communities on surfaces, such as the surfaces of medical implants and host organisms. The microbes in them, are embedded in a self-produced extracellular matrix (ECM)^1^ consisting of proteins^2^, polysaccharides^3^ and/or extracellular DNA (eDNA)^4^. The ECM has diverse functions to maintain the structural integrity of the biofilm and adapt to surrounding environments^5^. The resistance it confers to antimicrobial agents and host immune systems^6^ is a deleterious property, causative of various chronic human infectious diseases, including periodontal disease, bacteremia, pneumonia and meningitis. Biofilm-embedded bacteria cooperate and communicate with each other^5^. Thus, biofilms act as multi-cellular organisms, allowing microbes to survive in various environments and attack their host organisms^1^,^6^. Biofilms also cause familiar problems, such as those encountered in the maintenance of drinking water distribution systems.

Direct observation of the structure of biofilms is essential to understand their development and functions. Generally, optical microscopy (OM) is employed to observe bacterial cells or macro-organelles, and confocal laser scanning microscopy (CLSM) has made a large contribution to biofilm research^7^. Using fluorescence probes, CLSM can visualise the entire expanse of a biofilm, describe its overall structure and localize ECM constituents. However, the resolution of diffraction-limited OM is restricted to approximately 200 nm. Super-resolution OM overcomes this limitation^8^,^9^,^10^,^11^, and is providing exciting information about biofilms^12^. Nevertheless, this method images special fluorescence probes, and the visualisation of the surrounding structures is still awaited for the comprehensive understanding of biofilms.

Conventional electron microscopy (EM) has been widely used to image biofilms at sub-nanometre resolution^13^. However, the sample is under vacuum, making pretreatments that might affect the soft and hydrophilic structure of biofilms (e.g., dehydration) basically unavoidable. Recently, atmospheric scanning electron microscopy (ASEM) was developed to observe biological samples at atmospheric pressure^14^. ASEM allows an inverted SEM to observe a wet sample from below while an OM simultaneously observes it from above^15^. Cells can be cultured, fixed and imaged in the specialized sample dish (ASEM dish) at atmospheric pressure^16^,^17^. The dish has an electron transparent, 100 nm-thick silicon nitride (SiN_x_ is inevitably oxidized by the fabrication process, yielding SiN_x_O_y_^18^) film window in its base to allow SEM^14^,^15^, and holds a few millilitres of solution, enabling direct cell culture under stable conditions (Fig. 1). Importantly, at 30 kV acceleration voltage ASEM observes the 2–3 μm-thick specimen regions directly above the film, allowing structures near the film to be visualised at 8 nm resolution. In addition, the time-consuming sample preparation generally required for immuno-EM is not required for immuno-ASEM^16^,^19^ and the effort involved is comparable to that for immuno-OM.

In this study, we observed biofilms formed by a clinically important Gram-positive pathogen, *Staphylococcus aureus,* and a model Gram-negative bacterium, *Escherichia coli*. In combination with several labelling methods, ASEM could visualise the delicate structure of these biofilms and the fine structure of ECM components at the interface between the bacteria and the solid surface, in liquid (Fig. 1). Our results provide important information that will help to visualise biofilms immersed in solution by EM.

## Results

### Heavy metal staining of biofilms for ASEM

**Figure 1:**
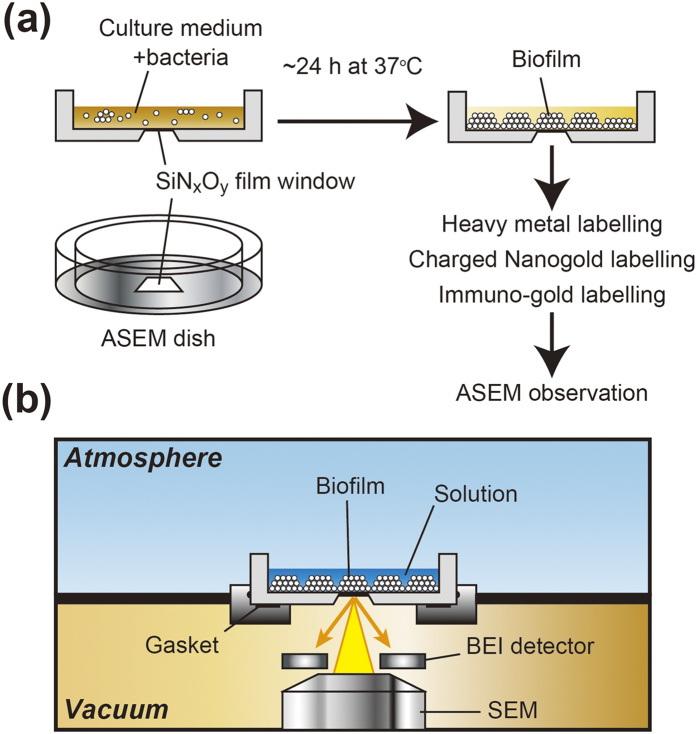
Outline of ASEM observation of staphylococcal biofilms in solution. (**a**) Bacteria were grown in an appropriate medium in the removable, 35-mm ASEM dish with SiN_x_O_y_ film window at the centre of its base. After the indicated incubation time at 37 °C, biofilms were fixed with 1% glutaraldehyde (GA) and 4% paraformaldehyde (PFA) for heavy metal-labelling and charged Nanogold-labelling or only with 4% PFA for immuno-labelling. After the indicated labelling procedures, the solution in the ASEM dish was replaced by a radical scavenger solution (10 mg/ml ascorbic acid or 10 mg/ml glucose). (**b**) Diagram of the ASEM showing the inverted SEM, the detector and the specimen dish, which separates the atmosphere (above) and the column vacuum (below). The electron beam passes through the window and is projected up onto the biofilms immersed in solution, penetrating them to a depth of 2–3 μm. Backscattered electrons (BSE) are captured by a BSE imaging (BEI) detector.

**Figure 2:**
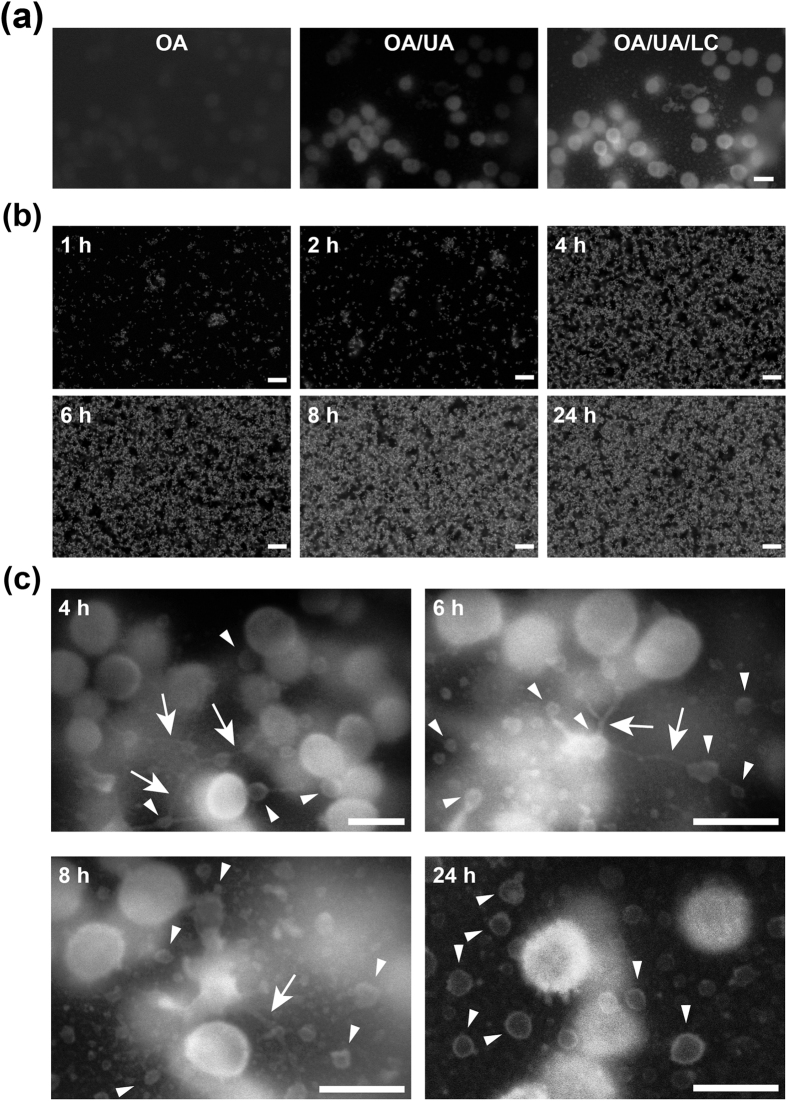
Heavy metal staining of *S. aureus* biofilms and their monitored formation. (**a**) *S. aureus* MR23 biofilm formed on an ASEM dish and, after 24 h, sequentially stained with heavy metals in the order: osmic acid (OA), uranyl acetate (UA), and lead citrate (LC). The ASEM images were recorded from the same area after each staining step. (**b**) Time course of biofilm formation on ASEM dishes. At the indicated time points, biofilms were stained with OA/UA/LC and observed by ASEM. (**c**) Higher magnification ASEM images of an MR23 biofilm at the indicated culture times. Arrows and arrowheads indicate filamentous and spherical structures, respectively. Scale bars, 1 μm in (**a,c**), and 10 μm in (**b)**.

Here we report staining protocols developed to allow the detailed visualisation of biofilms by ASEM (Fig. 1) and the information acquired by their use. Heavy metal stains with the ability to readily permeate fixed cells and stain specific biological molecules, such as proteins, lipids and nucleic acids, have been widely developed for EM. Therefore, we expected that these stains should be also applicable to thick biofilms. We tested the ability of osmic acid (OA), uranyl acetate (UA) and lead citrate (LC) to stain 24-h biofilms of a clinically isolated strain of methicillin-resistant *S. aureus* (MRSA), MR23, which produces robust proteinaceous biofilms (Supplementary Table 1)^20^,^21^,^22^. ASEM imaging at 30 kV revealed that sequential staining with OA, UA, and LC drastically improved image contrast (Fig. 2a). Using this method, biofilm development was visualised over time (Fig. 2b). The number of surface-attached cells increased with the incubation period, and the proliferation pattern correlated well with quantification data obtained by a conventional crystal violet (CV)-staining method (Supplementary Fig. 1), indicating that the heavy metal stain permeated the biofilms. ASEM images of 24-h biofilms in an aqueous environment revealed that bacterial cells do not align in close proximity to each other at the bottom of biofilms (Fig. 2a) and that there are cell-free regions, which are probably so-called water channels (Supplementary Fig. 2a,c,e). Importantly, air-drying biofilms induced abnormal cell aggregation on the solid surface (Supplementary Fig. 2b,d,f), indicating that ASEM of wet samples is required to visualise native biofilm structure.

### ASEM visualisation of the development of fibrillar nanostructures and vesicles in biofilms

**Figure 3:**
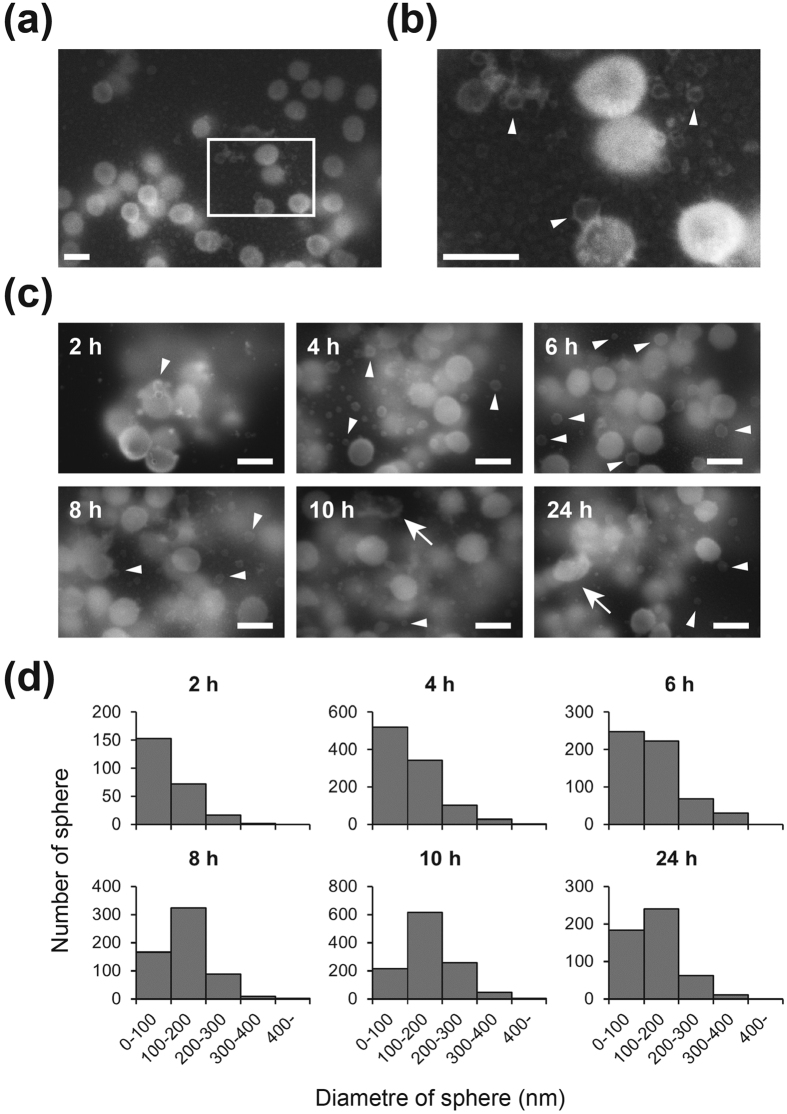
Spherical nanostructures within *S. aureus* biofilms. (**a**) ASEM image of an 8-h old *S. aureus* MR23 biofilm stained with OA/UA/LC. (**b**) Higher magnification image of the white rectangle in (**a**) revealing the presence of spherical nanostructures (arrowheads). (**c**) Production of spherical nanostructures (arrowheads) at the indicated time points during biofilm development. Broken bacterial cells (arrows) were also sometimes observed. (**d**) Histograms showing the number of spherical nanostructures with diameters in the indicated ranges at the indicated time points during biofilm development; as membrane vesicles (MVs) could only be observed by ASEM, the cultures were fixed at various time points, and arbitrary areas were imaged for manual statistical analysis. Scale bars, 1 μm.

**Figure 4:**
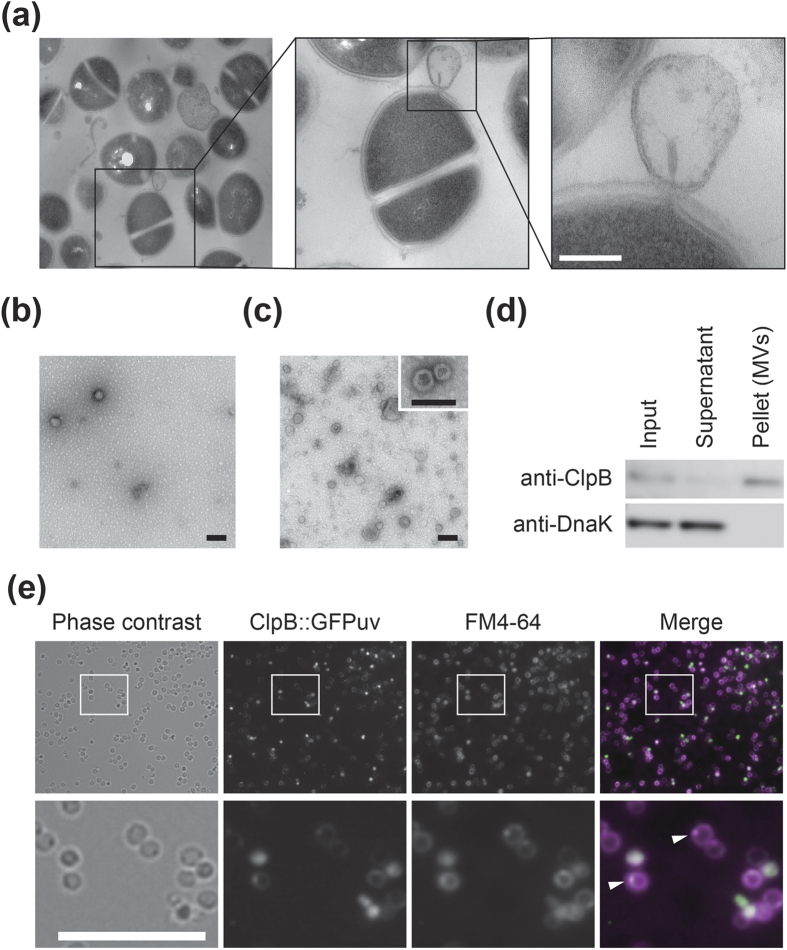
Observation of membrane vesicles produced by *S. aureus*. (**a**) Production of membrane vesicles (MVs) within 4-h biofilms of MR23 observed by TEM. Epon812 embedded biofilm was thin-sectioned serially and imaged. Higher magnification images of the black rectangles are also shown. (**b**,**c**) Isolated MVs from planktonic (**b**) and biofilm cultures (**c**) observed by negative stain TEM. A higher magnification image is shown in the inset of (**c**). (**d**) Immunoblots of isolated membrane vesicles. Input, supernatant, and pelleted fractions obtained by ultracentrifugation, were separated by SDS-PAGE and the separated proteins were blotted onto a PVDF membrane. The cytoplasmic molecular chaperones ClpB and DnaK were detected using anti-ClpB and anti-DnaK antibodies, respectively. While DnaK was found in the supernatant, excreted ClpB mainly present the membrane pellet. (**e**) Images of the ClpB::GFP_uv_ translational fusion produced from the genome of MR23. Membranes were stained with FM4-64 for the fluorescence microscopy. Phase contrast, green fluorescence (GFP_uv_), and red fluorescence (FM4-64) images are displayed in gray scales. The merged fluorescence image is also shown. Green: GFP_uv_; magenta: FM4-64. Lower panels: Higher magnification images of the white rectangles, documenting the secretion of ClpB in vesicles. Arrowheads indicate ClpB::GFP_uv_/FM4-64-positive spherical nanostructures. Scale bars, 100 nm in (**a–c**), 10 μm in (**e**).

Higher magnification ASEM images revealed that presence of nanostructures in 4- to 24-h biofilms of MR23 (Figs 2c and 3a,b). These included small spheres and fibrils. Careful observation also provided clear pictures of spheres associated with bacterial cells, and seem to be snapshots of the budding of membrane vesicles (MVs) (Fig. 3b). In agreement, when imaged by conventional SEM the cells were seen to have a rough-surface (Supplementary Fig. 3a–d). As demonstrated by time course images, the diameter of the spheres increased during biofilm development, and spheres with diameters ranging from 100 to 200 nm became major populations (Fig. 3c,d). Broken bacterial cells were also observed in 10- to 24-h biofilms (Fig. 3c). Serial thin-sectioning transmission electron microscopy (TEM) also captured snapshots of budding MVs, and indicated the presence of cytoplasmic substances within them (Fig. 4a, Supplementary Movies S1–S6). Next we isolated MVs from supernatants of planktonic and biofilm cultures. TEM images revealed that more MVs were produced under biofilm conditions than under planktonic conditions (Fig. 4b,c). The observed spherical structures were stained by the fluorescence-membrane probe FM4-64 and disappeared after detergent treatment, confirming their assigned identity (Supplementary Fig. 4). Sodium dodecyl sulphate-polyacrylamide gel electrophoresis (SDS) and Western blotting indicated that cytoplasmic proteins, such as the molecular chaperone ClpB, existed in the MV fractions (Fig. 4d). The presence of these cytoplasmic cargos was confirmed by fluorescence microscopy using the ClpB::GFP_uv_ translational fusion (Fig. 4e) and GFP_uv_ (Supplementary Fig. 5) derived from the *S. aureus* genome and plasmid, respectively. Collectively, these results suggest that cytoplasmic macromolecules, including proteins, are excreted via an MV-dependent pathway, as well as by cell lysis and uncharacterized transporters (Supplementary Fig. 6). MVs might also mediate the delivery of toxins, such as hemolysin (Supplementary Fig. 7a) and coagulase (Supplementary Fig. 7b), from the microbes within biofilms, as previously reported for various bacteria^23^,^24^,^25^.

### Charged Nanogold labelling of fibrils and flagella in biofilms

**Figure 5:**
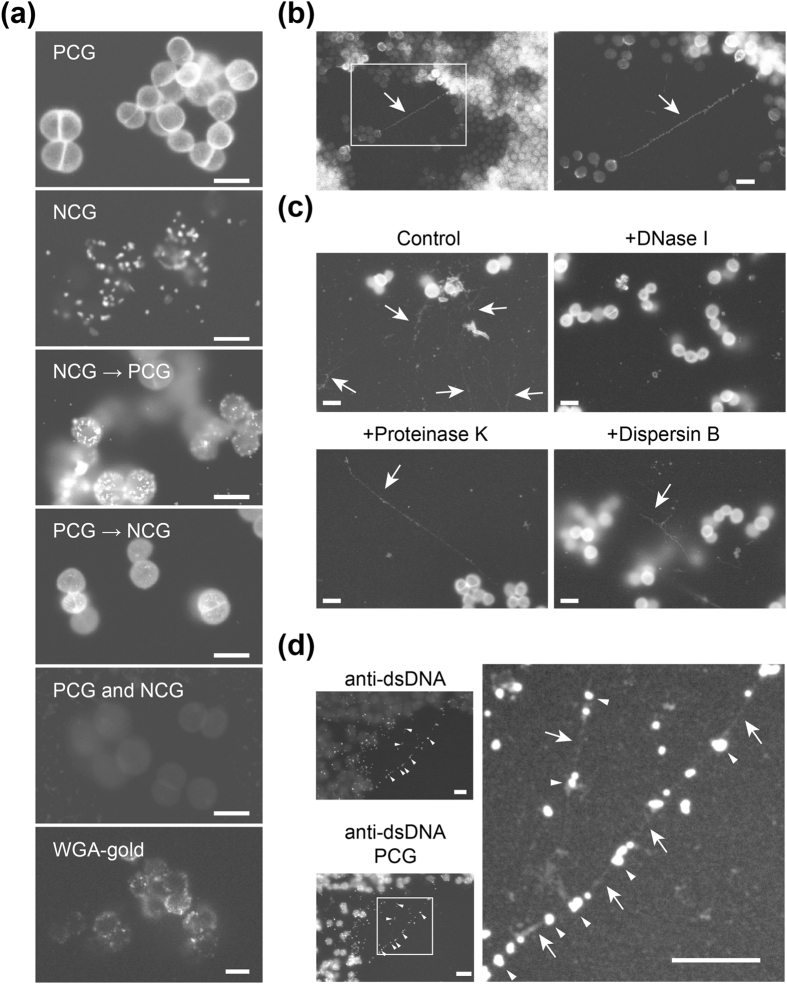
Charged Nanogold-labelling of biofilm matrix components. (**a**) Two-hour biofilms of *S. aureus* SH1000 were labelled with positively charged Nanogold (PCG), negatively charged Nanogold (NCG), or colloidal gold-conjugated wheat germ agglutinin (WGA-gold). PCG and NCG were used sequentially or, if required, simultaneously, as indicated. After gold enhancement, specimens were observed by ASEM. (**b**) Four-hour biofilms of *S. aureus* MR10 labelled with PCG, gold-enhanced, and observed by ASEM. A higher magnification image of the white rectangle is shown on the right. The arrow indicates a PCG-positive fibril. (**c**) *S. aureus* MR10 biofilms were grown in BHIG medium supplemented with, or without, the indicated enzymes for 4 h at 37 °C, labelled with PCG, and observed by ASEM. Arrows mark PCG-positive fibrillar structures. (**d**) Four-hour biofilms of MR10 were labelled with anti-dsDNA mouse IgG primary antibody and colloidal gold-conjugated anti-mouse IgG secondary antibody (anti-dsDNA). The biofilms were subsequently counter-stained with PCG (anti-dsDNA/PCG). A higher magnification image of the white rectangle is shown on the right. Arrowheads and arrows mark linearly aligned colloidal gold particles and PCG-positive fibrillar structures, respectively. Scale bars, 1 μm.

Positively charged Nanogold (PCG)-labelling is an efficient and rapid method used to visualise the surfaces of biological samples, such as bacteria and cultured animal cells, exploiting their net negative surface charges^26^,^27^. Here, we compared the ability of PCG and recently available negatively charged Nanogold (NCG) to label biofilms produced by *S. aureus* SH1000, which harbour ECM mainly comprised of extracellular polysaccharides^21^ (Supplementary Fig. 3e–h). PCG visualised biofilm cell surfaces clearly (Fig. 5a), indicating that the surface of *S. aureus*, which is composed of peptidoglycans, teichoic acids and proteins, has a net negative charge. In contrast, NCG formed high contrast dots on the cell surfaces but did not stain them entirely. The alignment patterns of the bright dots resemble those observed by lectin-labelling using a colloidal gold-conjugated wheat germ agglutinin (WGA-gold; Fig. 5a), suggesting that some of them correspond to polysaccharides and/or glycoproteins, both of which contain N-acetylglucosamine residues^28^,^29^ and, thus, interact with WGA. This is the first observation of bacterial samples labelled with NCG. Sequential labelling with NCG and then PCG resulted in combined labelling, while labelling with PCG and then NCG resulted in similar or lower contrast images than those obtained with PCG alone. Simultaneous labelling with PCG and NCG was inefficient, probably because the net charge of one masked that of the other. Collectively, these results indicate that PCG-labelling is the simplest and most efficient of the five Nanogold methods tested, resulting in high contrast images of *S. aureus* biofilms.

PCG-labelling was further applied to visualise biofilms formed by another *S. aureus* strain, MR10, which also produces robust biofilms harbouring ECM mainly comprised of extracellular polysaccharides^21^. Fibrils connecting MR10 cells were apparent (Fig. 5b). Similar structures were observed in other extracellular polysaccharide-rich biofilms of *Staphylococcus epidermidis* SE4^22^ by PCG-labelling and ASEM (Supplementary Fig. 8). Investigating MR10 biofilms with the fluorescent probe Alexa 488-conjugated WGA (WGA-Alexa 488) revealed that fibrils connected bacterial cells and were degraded by polysaccharide-hydrolysing enzyme dispersin B^22^ (Supplementary Fig. 9). However, we could not conclude that PCG-positive fibrils visualised by ASEM were in fact polysaccharide networks as the ECM of MR10 biofilms also harbours DNA^22^, a ubiquitous and pivotal component in diverse bacterial biofilms^4^. To assess this precisely, MR10 biofilms were formed in the presence or absence of ECM-degrading enzymes, including DNase I, proteinase K, and dispersin B and imaged from below by ASEM. PCG-positive *S. aureus* fibrils became invisible in the presence of DNase I, but were still observed in biofilms formed in the presence of dispersin B and proteinase K (Fig. 5c). Immuno-labelling with anti-double strand DNA (dsDNA) primary antibody and colloidal gold-conjugated secondary antibody resulted in linearly aligned gold particles, and counter-labelling with PCG enhanced the contrast of the regions between them revealing fibrils (Fig. 5d). In contrast, when primary antibody-labelling was omitted, such gold particles was not observed (Supplementary Fig. 10). These results indicate that the *S. aureus* fibrils observed by PCG-labelling and ASEM were DNA fibrils present at the bottom of the biofilms, whereas polysaccharides, main ECM components, might cover the top surface of the biofilms as observed by conventional SEM (Supplementary Fig. 3e–h).

**Figure 6:**
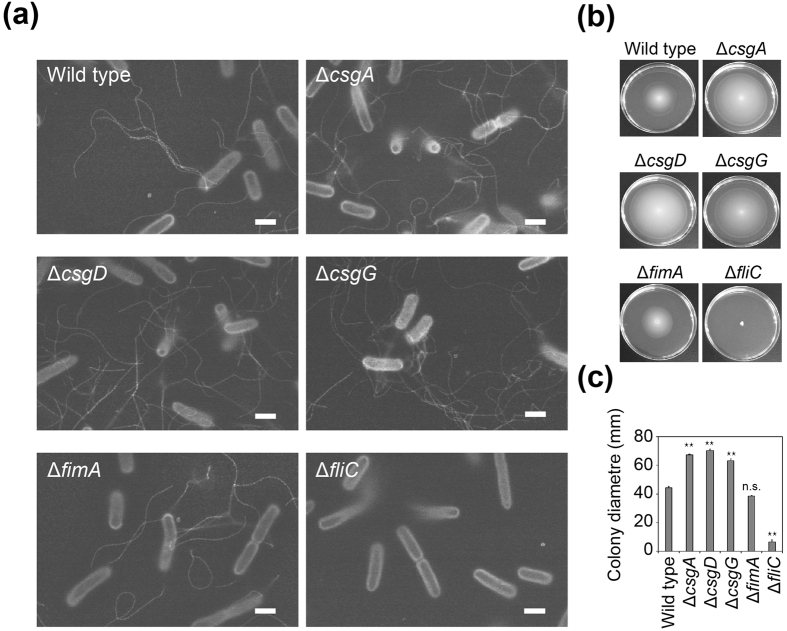
Visualisation of flagella by PCG-labelling ASEM. (**a**) The indicated *E. coli* strains were labelled with PCG and observed in liquid by ASEM. Flagella were prominent. (**b**) Swimming activity of the *E. coli* strains on 0.3% soft agar plates. (**c**) Estimated diameters of the *E. coli* colonies in (**b)**. The means and standard deviations of results from at least three independent experiments are shown. **P < 0.01; n.s., not significant. Scale bars, 1 μm.

Flagella are thought to be involved in surface attachment of bacteria at the initial stage of biofilm development^30^,^31^. PCG-labelling is potentially useful for visualising flagella as reported previously^26^. Here, we visualised flagella more carefully for various strains of *E. coli*, i.e., the K-12 wild type^32^ and the isogenic mutants: Δ*fliC*, Δ*csgA*, Δ*csgD*, Δ*csgG*, and Δ*fimA*. The *fliC* gene is involved in the biosynthesis of flagella^33^; the *csgA, csgD*, and *csgG* genes are required for the production of curli, the extracellular functional amyloid that is crucial for biofilm formation^34^; the *fimA* gene encodes the type I pili subunit^35^. Delicate spiral fibrillar structures were observed in all of the strains examined, except the Δ*fliC* mutant (Fig. 6a, Supplementary Fig. 11). Interestingly, the number of flagella per cell for Δ*csgA*, Δ*csgD*, and Δ*csgG* was more than for the wild type and Δ*fimA* (Fig. 6a, Supplementary Fig. 11). This observation was highly correlated with the motility measured on a soft agar plate (Fig. 6b,c), suggesting that curli production negatively regulates flagellum production. Cross-regulation between flagella and curli biosynthesis is mediated by the flagellar master regulator FlhDC, the stationary phase sigma factor σ^S^ (RpoS) and the key biofilm regulator CsgD. All have previously been shown to act as major hubs regulated by small RNAs^36^,^37^. According to the finding that the *fliE* and *fliFGHIJK* operons for flagellum formation are directly repressed by CsgD^36^, increased production of flagella in the Δ*csgD* strain is understandable. On the other hand, the mechanism(s) causing increased flagellum production in the Δ*csgA* and Δ*csgG* strains is unclear. Curli themselves might induce cell envelope alterations, leading to the negative regulation of FlhDC by the RscCDB His-Asp phosphorelay system^38^.

### Immuno-labelling of proteins in *Staphylococcus aureus* biofilms

**Figure 7:**
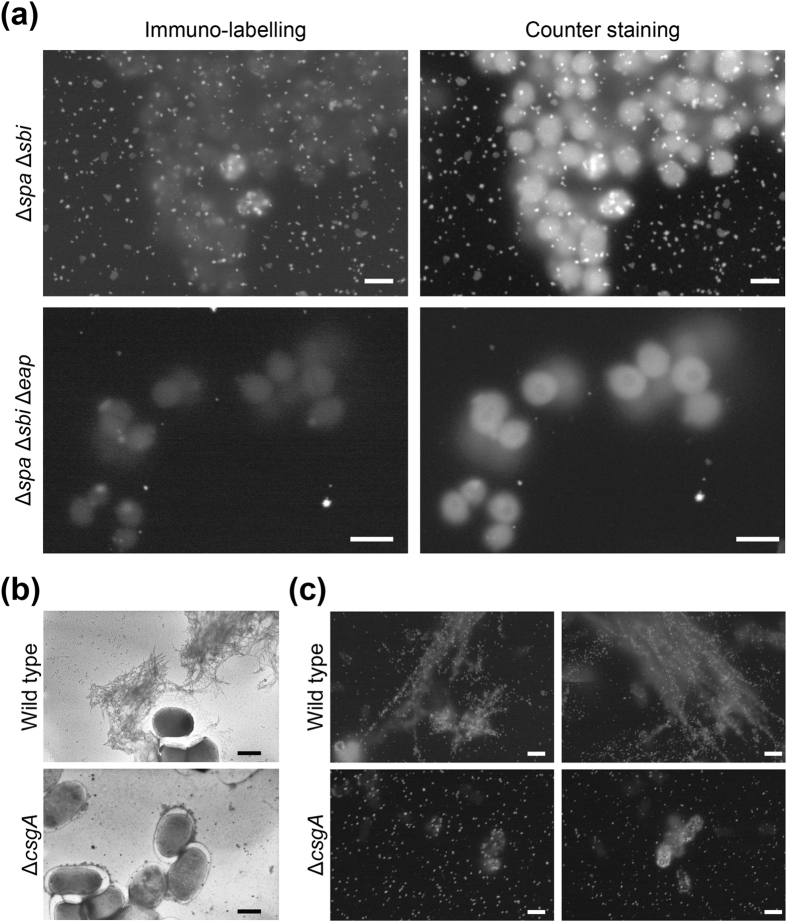
Immunogold-labelling to detect matrix components in biofilms. (**a**) Immuno-labelled biofilm cells of the MR23 Δ*spa* Δ*sbi* mutant (top), which was used to prevent nonspecific binding of IgGs to Spa and Sbi, and the similarly treated MR23 Δ*spa* Δ*sbi* Δ*eap* mutant (bottom), which was used as a negative control. The same areas are shown in the left (immuno-labelling) and the right (counter staining) panels. (**b,c**) Curli production in the wild type and the Δ*csgA* strains for 7 days at 25 °C observed by negative stain TEM after air-drying (**b**) and in solution by immuno-labelled ASEM (**c**). Curli were labelled with anti-curli rabbit IgG primary antibody and Nanogold-conjugated secondary antibody for the ASEM. Two typical images of these strains are shown. Scale bars, 1 μm.

The ease with which immuno-labelling can be achieved is a great advantage of ASEM over conventional EM^16^,^27^, however, the penetration of antibodies is critical for observing biofilms. Moreover, the presence of several IgG-binding proteins, including staphylococcal protein A (Spa) and the second IgG-binding protein (Sbi) in *S. aureus* biofilms (Supplementary Fig. 12a–c), prevents the immuno-detection of some proteins of interest^22^. We, therefore, constructed *S. aureus* mutants lacking the *spa* and/or *sbi* gene(s). Wild type and single deletion mutants displayed non-specific IgG-binding, but this was abolished in the double knockouts (Supplementary Fig. 12d). Of note, all mutants formed biofilms at the level of the wild type (Supplementary Fig. 12e). Using anti-Eap primary antibody and Nanogold-conjugated goat anti-rabbit Fab’, the extracellular adherence protein (Eap) was labelled in 2-h thin biofilms formed by the Δ*spa* Δ*sbi* strain, and cells were subsequently, counter stained by a modified National Center for Microscopy and Imaging Research (NCMIR) method (Supplementary Fig. 13)^39^. ASEM revealed gold particles localized on the surface of Δ*spa* Δ*sbi* cells and around them (Fig. 7a, upper panels), as previously demonstrated by fluorescence microscopy^21^. However, only few signals from gold particles were observed for the control Δ*spa* Δ*sbi* Δ*eap* strain (Fig. 7a, lower panels). Fab fragments of primary antibodies and colloidal gold-conjugated Spa were also useful for immuno-labelling cell surface proteins, such as Spa and the excreted cytoplasmic molecular chaperones ClpB and DnaK, both of which were identified in biofilm matrices of *S. aureus*^21^ (Supplementary Figs 14 and 15). In both experiment series, counter staining by the modified NCMIR method enhanced the contrast of bacterial cells (Fig. 7a) more than counter staining with OA and UA (Supplementary Figs 14 and 15).

### Straight curli in wet biofilms imaged by immuno-labelling ASEM

We next visualised curli produced in *E. coli* colony biofilms using antibodies raised against purified curli^22^. Curli, but neither type I pili nor flagella, were crucial for biofilm formation in *E. coli* under the conditions tested (Supplementary Fig. 16). The importance of flagella for biofilm development depends on the culture media and exact conditions used^30^,^40^ and is still being debated^41^. We did not notice any differences in the number of surface-attached cells for the six strains under the conditions tested (Supplementary Fig. 11). Interestingly, TEM images of negatively stained air-dried curli revealed winding fibrils (Fig. 7b), as reported previously^34^, whereas, immuno-ASEM revealed linearly aligned gold-particles (Fig. 7c), indicating that curli present as straight filaments in an aqueous environment. Thus, air-drying for conventional EM might affect their morphology. Together the results presented show that immuno-ASEM in aqueous solution can be used to visualise the localization and structure of matrix components in biofilms.

## Discussion

Here, we report the first observation of bacterial biofilms immersed in an aqueous solution by ASEM (Fig. 1). In combination with several labelling methods, including heavy metal-labelling (Figs 2 and 3), charged Nanogold-labelling (Figs 5 and 6), and immuno-labelling (Fig. 7), ASEM was able to visualise delicate hydrophilic nano-structures of the biofilm-surface interface (Supplementary Fig. 2). This was the case for various types of biofilms produced by Gram-positive cocci (Figs 2,3,5 and 7a) and Gram-negative bacilli (Figs 6 and 7c), suggesting the applicability of this cutting-edge technique to diverse bacterial biofilms. As ASEM can visualise various eukaryotic cells^14^,^15^,^16^,^17^,^26^,^27^,^39^,^42^, these should include biofilms formed by fungi.

Environmental cell (EC)-EM^43^ and environmental EM (EEM)^44^ were also developed to observe biological samples in aqueous solution. For EC-TEM, a capsule with two electron transparent film, now known as an environmental cell (EC) was developed and improved^45^,^46^,^47^, and has diverged to EC-SEM^48^. ECs can hold aqueous and gaseous samples, and are placed inside the microscope. However, their small volume (<20 μl) and dimensions probably making immuno-labelling experiments relatively difficult. EEM requires pressure-limiting apertures to allow the observation of samples in a low pressure gas by TEM and SEM. The hydration of the specimens is maintained for a limited period due to evaporation. Compared to EC-EM^43^,^45^,^46^,^47^,^48^,^49^,^50^ and EEM^44^, ASEM is easy to use and high throughput.

Atomic force microscopy (AFM) is also a powerful tool for the high-resolution imaging and analysis of biofilms, due to its capacity for imaging with a higher resolution than OM in both air and liquid^51^. Since it can visualise samples without fixing, labelling and staining, non-invasive imaging can be performed on surfaces in their native states. The force measurement capacity of AFM can provide unique insights into bacteria-bacteria and bacteria-surface interactions^52^. On the other hand, there are limitations of AFM for the study of biofilms. Imaging of rough surface biofilms with a large difference in height often become difficult. Similar to conventional SEM, AFM obtains top surface images of biofilms but is not able to visualise the interface structure between bacteria and surfaces^51^. Combining AFM with other techniques including ASEM is a way to study biofilm structures and physiology.

Among the labelling methods used for ASEM in our study, PCG-labelling was the most efficient, simplest and fastest, sample preparation taking less than half an hour and allowing both bacterial cells and extracellular structures to be visualised. PCG-labelling was also useful to counter-stain the immunogold-labelled samples (Fig. 5d). However, the permeation of PCG was insufficient for thick biofilms, probably due to the presence of a large amount of negatively charged molecules in biofilms, which would trap PCG particles. In this case, sequential heavy metal-labelling (OA/UA/LC) provided high contrast images and proved to be a powerful method (Figs 2 and 3), even though it requires a longer labelling/washing time. Immuno-labelling was also easy for *E. coli* biofilms (Fig. 7c). However, specific labelling in *S. aureus* biofilms was difficult due to the presence of the IgG-binding proteins, Spa and Sbi. As we demonstrate, this problem can be overcome in three ways: (i) by the use of a Δ*spa* Δ*sbi* strain (Fig. 7a); (ii) by labelling with Fab fragments of primary antibodies (Supplementary Fig. 14); (iii) by using colloidal gold-conjugated Spa instead of the gold-conjugated secondary antibody (Supplementary Fig. 15). These methods were effective for thin biofilms formed in a few hours (2–4 h), but not for thick biofilms due to the inability of antibodies to permeate them, like PCG. The modified NCMIR method proved to be an excellent way to counter stain immuno-labelled biofilms (Fig. 7a), and was superior to other staining methods (Supplementary Figs 14 and 15).

The ASEM system has an inverted SEM below the sample and an OM above it, providing a large potential for correlative OM/EM imaging^16^,^17^. CLSM can visualise structure of biofilms at higher resolution than conventional fluorescent microscopy^12^, and a recent advance including multiphoton confocal microscopy, is providing exciting results in biofilm biology^7^. These advanced OM may compensate the weak point of the present ASEM that can visualise only 2–3 μm thick specimen regions directly above the SiN_x_O_y_ film. The development of an ASEM/CLSM hybrid machine is feasible, and would allow the 3D structure of biofilms and their ultrastructure at the bacteria-surface interface to be visualised. Our results provide a new methodology for observing biofilms, and include several findings of biological significance, as mentioned above. ASEM should shed light on unresolved questions, and may also lead to a new research direction in biology.

## Methods

### Bacterial strains and plasmids

The bacterial strains and plasmids used in this study are listed in Supplementary Table 1. Detailed procedures for the construction of *S. aureus* mutant strains and culture conditions are described in the Supplementary Methods.

### Biofilm formation

Staphylococcal biofilms were formed in brain heart infusion (BHI) medium (Becton Dickinson) supplemented with 1% glucose (BHIG) in ASEM dishes (JEOL, Tokyo, Japan) (Fig. 1), 96-well plates (Corning, Corning, NY, USA), 35-mm plastic dishes (Nunc, Tokyo, Japan), and glass bottom dishes (Matsunami glass, Tokyo, Japan) over the indicated time periods at 37 °C. Biofilms formed in ASEM dishes were fixed, stained, labelled, and imaged by ASEM. Biofilms formed in 96-well plates were stained with 0.2% crystal violet (CV) and quantified by measuring the absorbance at 595 nm with a micro titre plate reader (Infinite F200 Pro, Tecan, Männedorf, Switzerland). Biofilms formed in 35 mm dishes were stained as previously reported^20^. Biofilms produced in glass bottom dishes were used for indirect immunofluorescence microscopy and lectin-labelling.

*E. coli* biofilms were formed in YESCA medium in 96-well plates for 7 days at 25 °C and were quantified as previously reported^32^. *E. coli* colony biofilms were formed on YESCA plates for 7 days at 25 °C^22^, and were imaged by ASEM and TEM^32^.

### Fixation

Biofilms were fixed with 4% paraformaldehyde (PFA) and 1% glutaraldehyde (GA) for 10 min at room temperature for heavy metal staining and charged Nanogold labelling. Biofilms were fixed with 4% PFA for 10 min at room temperature for immuno-labelling. After immuno-labelling, biofilms were further fixed with 1% GA for 10 min at room temperature, to stabilize the antibody interactions prior to gold enhancement and/or counter staining with heavy metals.

### Heavy metal staining

Biofilms on the ASEM dish were treated twice with 2% OA for 20 min, then with 2% UA for 25 min, and subsequently washed with double distilled water (DDW). If required, the biofilms were further stained with 0.4% LC in 0.4% sodium hydroxide for 5 min.

### Modified NCMIR staining method

Immuno-labelled biofilms on the ASEM dish were stained using a slight modification of the NCMIR method developed by the Ellisman’s group^53^ as recently reported^39^.

### Charged Nanogold labelling

For charged Nanogold-labelling, bacteria on the ASEM dish were incubated with 6 μM positively and/or negatively charged 1.4 nm Nanogold solution (Nanoprobes, Stony Brook, NY, USA) for 20 min at room temperature as previously reported^26^,^42^. After washing with DDW, the size of the gold particles was increased by gold enhancement using GoldEnhance-EM (Nanoprobes) for 10 min at room temperature, followed by washing with DDW. The bacteria were imaged by ASEM.

### Immuno-labelling

PFA-fixed staphylococcal biofilms were incubated with 5% skimmed milk and 1% goat serum (Sigma, St. Louis, MO, USA) in buffer A [40 mM HEPES (pH 7.4) and 150 mM NaCl] for 1 h at room temperature. PFA-fixed *E. coli* cells were incubated with 5% skimmed milk in buffer A for 1 h at room temperature. Detailed information of the immuno-labelling is given in the Supplementary Methods.

### Gold-conjugated lectin-labelling

For lectin labelling, one-hour biofilms of *S. aureus* SH1000 were fixed with 4% PFA and 1% GA for 10 min at room temperature, washed with PBS, and incubated with 30-nm colloidal gold-conjugated wheat germ agglutinin solution (WGA-Gold, EY Laboratories, 1/5 dilution in PBS) for 30 min at 37 °C. After washing with PBS, WGA-Gold was fixed with 1% GA for 10 min at room temperature, followed by washing with DDW. The biofilms were imaged by ASEM.

### ASEM imaging

ASEM images were recorded using the ClairScope ASEM system (JASM-6200, JEOL, Ltd, Tokyo, Japan) (Fig. 1)^14^ as described in the Supplementary Methods.

### TEM imaging

Thin sections of *S. aureus* MR23 biofilm cells, the MV fractions isolated from *S. aureus* biofilm and planktonic cultures, and *E. coli* colony biofilms^32^ were observed by TEM as described in the Supplementary Methods.

### Fluorescence microscopy

*S. aureus* MR23 cells, expressing the ClpB::GFP_uv_ translational fusion (Supplementary Table 1) or GFP_uv_ from the plasmid pP1GFP_uv_^21^, were cultured in BHIG medium for 24 h at 37 °C. Aliquots (5 μl) of the cultures were spotted on a glass slide under a cover slip. Specimens were observed using a fluorescence microscope (Nikon, Tokyo, Japan) equipped with B2 (excitation, 450–490 nm; barrier, 520 nm), UV-1A (excitation, 360–370 nm; barrier, 420 nm) and G2A (excitation, 510–560 nm; barrier, 590 nm) filters.

Membranes and DNA were stained with FM4-64 (Life Technologies) and DAPI (Dojindo Laboratory, Kumamoto, Japan), respectively, as recently reported^54^. Extracellular polysaccharides of *S. aureus* were labelled with Alexa Fluor 488-conjugated WGA (WGA-Alexa 488, Life Technologies) and observed by fluorescence microscopy as previously reported^22^.

### Congo red (CR)-binding assay

Curli produced by *E. coli* strains were analysed on YESCA plate containing 2% agar, 10 μg/ml Congo red, and 10 μg/ml Coomassie brilliant blue G-250 as recently reported^32^.

### Isolation of MVs

MVs were isolated from the supernatants of biofilm cultures and planktonic cultures of *S. aureus* MR23 grown in BHIG medium for 6 h at 37 °C. The 50-ml cultures were centrifuged at 10,000 × g for 25 min at 4 °C. The supernatants were passed through a 0.2 μm filter, and the resulting solutions were concentrated to less than 1 ml using an Amicon 100 K filter (Millipore). The solutions were ultracentrifuged at 150,000 × g for 3 h at 4 °C. The supernatants were collected. The pellets were re-suspended in 1 ml of 10 mM HEPES (pH 7.0) and 100 mM NaCl, and imaged by TEM. The supernatant and pellet (MVs) fractions were used for further experiments.

### SDS-PAGE and Western blotting

The concentrated supernatant of the biofilm culture and the supernatant and pellet fractions obtained by ultracentrifugation (see above) were analysed by SDS-PAGE, followed by Western blotting as described in the Supplementary Methods.

### Swimming assay

Bacterial swimming activity was examined on a soft agar plate. In parallel, flagella were visualised by ASEM. Detailed information is provided in the Supplementary Methods.

### Statistical analysis

The Student’s *t* test was used to assess biofilm formation and the swimming assays. A value of <0.05 was considered to indicate statistical significance. The means and standard deviations of the results from at least three independent experiments were also calculated.

## Additional Information

**How to cite this article**: Sugimoto, S. *et al*. Imaging of bacterial multicellular behaviour in biofilms in liquid by atmospheric scanning electron microscopy. *Sci. Rep.* **6**, 25889; doi: 10.1038/srep25889 (2016).

## Supplementary Material

Supplementary Information

## References

[b1] Costerton J. W., Stewart P. S. & Greenberg E. P. Bacterial biofilms: a common cause of persistent infections. Science 284, 1318–1322 (1999). 10334980 10.1126/science.284.5418.1318

[b2] Olsen A., Jonsson A. & Normark S. Fibronectin binding mediated by a novel class of surface organelles on *Escherichia coli*. Nature 338, 652–655 (1989). 2649795 10.1038/338652a0

[b3] Eighmy T. T., Maratea D. & Bishop P. L. Electron microscopic examination of wastewater biofilm formation and structural components. Appl. Environ. Microbiol. 45, 1921–1931 (1983). 6881965 10.1128/aem.45.6.1921-1931.1983 PMC242559

[b4] Whitchurch C. B., Tolker-Nielsen T., Ragas P. C. & Mattick J. S. Extracellular DNA required for bacterial biofilm formation. Science 295, 1487 (2002). 11859186 10.1126/science.295.5559.1487

[b5] Flemming H. C. & Wingender J. The biofilm matrix. Nature Rev. Microbiol. 8, 623–633 (2010). 20676145 10.1038/nrmicro2415

[b6] Davies D. Understanding biofilm resistance to antibacterial agents. Nat. Rev. Drug Discov. 2, 114–122 (2003). 12563302 10.1038/nrd1008

[b7] Neu T. R. & Lawrence J. R. Innovative techniques, sensors, and approaches for imaging biofilms at different scales. Trends Microbiol. 23, 233–242 (2015). 25623967 10.1016/j.tim.2014.12.010

[b8] Hell S. W. & Wichmann J. Breaking the diffraction resolution limit by stimulated emission: stimulated-emission-depletion fluorescence microscopy. Opt. Let. 19, 780–782 (1994). 19844443 10.1364/ol.19.000780

[b9] Gustafsson M. G. Nonlinear structured-illumination microscopy: wide-field fluorescence imaging with theoretically unlimited resolution. Proc. Natl. Acad. Sci. USA 102, 13081–13086 (2005). 16141335 10.1073/pnas.0406877102 PMC1201569

[b10] Betzig E. . Imaging intracellular fluorescent proteins at nanometer resolution. Science 313, 1642–1645 (2006). 16902090 10.1126/science.1127344

[b11] Rust M. J., Bates M. & Zhuang X. Sub-diffraction-limit imaging by stochastic optical reconstruction microscopy. Nat. Methods 3, 793–795 (2006). 16896339 10.1038/nmeth929 PMC2700296

[b12] Berk V. . Molecular architecture and assembly principles of *Vibrio cholerae* biofilms. Science 337, 236–239 (2012). 22798614 10.1126/science.1222981 PMC3513368

[b13] Marrie T. J., Nelligan J. & Costerton J. W. A scanning and transmission electron microscopic study of an infected endocardial pacemaker lead. Circulation 66, 1339–1341 (1982). 7139907 10.1161/01.cir.66.6.1339

[b14] Nishiyama H. . Atmospheric scanning electron microscope observes cells and tissues in open medium through silicon nitride film. J. Struct. Biol. 169, 438–449 (2010). 20079847 10.1016/j.jsb.2010.01.005

[b15] Nishiyama H. . Atmospheric scanning electron microscope system with an open sample chamber: configuration and applications. Ultramicroscopy 147, 86–97 (2014). 25062041 10.1016/j.ultramic.2014.06.001

[b16] Maruyama Y., Ebihara T., Nishiyama H., Suga M. & Sato C. Immuno EM-OM correlative microscopy in solution by atmospheric scanning electron microscopy (ASEM). J. Struct. Biol. 180, 259–270 (2012). 22959994 10.1016/j.jsb.2012.08.006

[b17] Hirano K. . Electron microscopy of primary cell cultures in solution and correlative optical microscopy using ASEM. Ultramicroscopy 143, 52–66 (2014). 24216127 10.1016/j.ultramic.2013.10.010

[b18] Zaluzec N. J. When is Si3N4 not Si3N4? When it is a low stress SiNx membrane window. Microsc. Microanal. 21 (Suppl 3), 959–960 (2015).

[b19] Sato C. . Rapid imaging of mycoplasma in solution using Atmospheric Scanning Electron Microscopy (ASEM). Biochem. Biophys. Res. Commun. 417, 1213–1218 (2012). 22226908 10.1016/j.bbrc.2011.12.111

[b20] Sugimoto S. . Cloning, expression and purification of extracellular serine protease Esp, a biofilm-degrading enzyme, from S*taphylococcus epidermidis*. J. Appl. Microbiol. 111, 1406–1415 (2011). 21974778 10.1111/j.1365-2672.2011.05167.x

[b21] Sugimoto S. . *Staphylococcus epidermidis* Esp degrades specific proteins associated with *Staphylococcus aureus* biofilm formation and host-pathogen interaction. J. Bacteriol. 195, 1645–1655 (2013). 23316041 10.1128/JB.01672-12 PMC3624567

[b22] Chiba A., Sugimoto S., Sato F., Hori S. & Mizunoe Y. A refined technique for extraction of extracellular matrices from bacterial biofilms and its applicability. Microb. Biotechnol. 8, 392–403 (2015). 25154775 10.1111/1751-7915.12155 PMC4408173

[b23] Wai S. N. . Vesicle-mediated export and assembly of pore-forming oligomers of the enterobacterial ClyA cytotoxin. Cell 115, 25–35 (2003). 14532000 10.1016/s0092-8674(03)00754-2

[b24] Vanhove A. S. . Outer membrane vesicles are vehicles for the delivery of *Vibrio tasmaniensis* virulence factors to oyster immune cells. Environ. Microbiol. 17, 1152–1165 (2015). 24919412 10.1111/1462-2920.12535

[b25] Thay B., Wai S. N. & Oscarsson J. *Staphylococcus aureus* alpha-toxin-dependent induction of host cell death by membrane-derived vesicles. Plos One 8, e54661 (2013). 23382935 10.1371/journal.pone.0054661 PMC3561366

[b26] Nishiyama H., Teramoto K., Suga M. & Sato C. Positively charged Nanogold label allows the observation of fine cell filopodia and flagella in solution by atmospheric scanning electron microscopy. Microsc. Res. Tech. 77, 153–160 (2014). 24343867 10.1002/jemt.22322

[b27] Kinoshita T. . Immuno-electron microscopy of primary cell cultures from genetically modified animals in liquid by atmospheric scanning electron microscopy. Microsc. Microanal. 20, 469–483 (2014). 24564988 10.1017/S1431927614000178

[b28] Mack D. . The intercellular adhesin involved in biofilm accumulation of *Staphylococcus epidermidis* is a linear beta-1,6-linked glucosaminoglycan: purification and structural analysis. J. Bacteriol. 178, 175–183 (1996). 8550413 10.1128/jb.178.1.175-183.1996 PMC177636

[b29] Hazenbos W. L. . Novel staphylococcal glycosyltransferases SdgA and SdgB mediate immunogenicity and protection of virulence-associated cell wall proteins. PLoS Pathog. 9, e1003653 (2013). 24130480 10.1371/journal.ppat.1003653 PMC3794999

[b30] O’Toole G. A. & Kolter R. Flagellar and twitching motility are necessary for *Pseudomonas aeruginosa* biofilm development. Mol. Microbiol. 30, 295–304 (1998). 9791175 10.1046/j.1365-2958.1998.01062.x

[b31] Pratt L. A. & Kolter R. Genetic analysis of *Escherichia coli* biofilm formation: roles of flagella, motility, chemotaxis and type I pili. Mol. Microbiol. 30, 285–293 (1998). 9791174 10.1046/j.1365-2958.1998.01061.x

[b32] Arita-Morioka K., Yamanaka K., Mizunoe Y., Ogura T. & Sugimoto S. Novel strategy for biofilm inhibition by using small molecules targeting molecular chaperone DnaK. Antimicrob. Agents Chemother. 59, 633–641 (2015). 25403660 10.1128/AAC.04465-14 PMC4291377

[b33] Kuwajima G., Asaka J., Fujiwara T., Fujiwara T., Node K. & Kondo E. Nucleotide sequence of the hag gene encoding flagellin of *Escherichia coli*. J. Bacteriol. 168, 1479–1483 (1996). 3536885 10.1128/jb.168.3.1479-1483.1986 PMC213667

[b34] Chapman M. R. . Role of *Escherichia coli* curli operons in directing amyloid fiber formation. Science 295, 851–855 (2002). 11823641 10.1126/science.1067484 PMC2838482

[b35] Klemm P. The *fimA* gene encoding the type-1 fimbrial subunit of *Escherichia coli*. Nucleotide sequence and primary structure of the protein. Eur. J. Biochem. 143, 395–399 (1984). 6147250 10.1111/j.1432-1033.1984.tb08386.x

[b36] Ogasawara H., Yamamoto K. & Ishihama A. Role of the biofilm master regulator CsgD in cross-regulation between biofilm formation and flagellar synthesis. J. Bacteriol. 193, 2587–2597 (2011). 21421764 10.1128/JB.01468-10 PMC3133154

[b37] Mika F. & Hengge R. Small Regulatory RNAs in the control of motility and biofilm formation in *E. coli* and *Salmonella*. Int. J. Mol. Sci. 14, 4560–4579 (2013). 23443158 10.3390/ijms14034560 PMC3634460

[b38] Francez-Charlot A. . RcsCDB His-Asp phosphorelay system negatively regulates the *flhDC* operon in *Escherichia coli*. Mol. Microbiol. 49, 823–832 (2003). 12864862 10.1046/j.1365-2958.2003.03601.x

[b39] Memtily N. . Observation of tissues in open aqueous solution by atmospheric scanning electron microscopy: applicability to intraoperative cancer diagnosis. Int. J. Oncol. 46, 1872–1882 (2015). 25707365 10.3892/ijo.2015.2905 PMC4383018

[b40] Prigent-Combaret C. . Developmental pathway for biofilm formation in curli-producing *Escherichia coli* strains: role of flagella, curli and colanic acid. Environ. Microbiol. 2, 450–464 (2000). 11234933 10.1046/j.1462-2920.2000.00128.x

[b41] Hengge R. Reply to “precedence for the structural role of flagella in biofilms”. mBio 4, e00245–00213 (2013). 23611911 10.1128/mBio.00245-13 PMC3631615

[b42] Murai T., Sato M., Nishiyama H., Suga M. & Sato C. Ultrastructural analysis of Nanogold-labeled cell surface microvilli in liquid by atmospheric scanning electron microscopy and their relevance in cell adhesion. Int. J. Mol. Sci. 14, 20809–20819 (2013). 24135874 10.3390/ijms141020809 PMC3821644

[b43] Abrams I. M. & McBain J. W. A closed cell for electron microscopy. Science 100, 273–274 (1944). 17746136 10.1126/science.100.2595.273

[b44] Danilatos G. D. Introduction to the ESEM instrument. Microsc. Res. Tech. 25, 354–361 (1993). 8400426 10.1002/jemt.1070250503

[b45] Evans J. E. . Visualizing macromolecular complexes with *in situ* liquid scanning transmission electron microscopy. Micron 43, 1085–1090 (2012). 22386621 10.1016/j.micron.2012.01.018 PMC9979698

[b46] Williamson M. J., Tromp R. M., Vereecken P. M., Hull R. & Ross F. M. Dynamic microscopy of nanoscale cluster growth at the solid-liquid interface. Nat. Materials 2, 532–536 (2003). 12872162 10.1038/nmat944

[b47] de Jonge N. & Ross F. M. Electron microscopy of specimens in liquid. Nat. Nanotechnol. 6, 695–704 (2011). 22020120 10.1038/nnano.2011.161

[b48] Thiberge S. . Scanning electron microscopy of cells and tissues under fully hydrated conditions. Proc. Natl. Acad. Sci. USA 101, 3346–3351 (2004). 14988502 10.1073/pnas.0400088101 PMC376183

[b49] Fukushima K., Katoh M. & Fukami A. Quantitative measurements of radiation damage to hydrated specimens observed in a wet gas environment. J. Electron Microsc. 31, 119–126 (1982). 6927401

[b50] de Jonge N., Peckys D. B., Kremers G. J. & Piston D. W. Electron microscopy of whole cells in liquid with nanometer resolution. Proc. Natl. Acad. Sci. USA 106, 2159–2164 (2009). 19164524 10.1073/pnas.0809567106 PMC2650183

[b51] Wright C. J., Shah M. K., Powell L. C. & Armstrong I. Application of AFM from microbial cell to biofilm. Scanning 32, 134–149 (2010). 20648545 10.1002/sca.20193

[b52] Beech I., Smith J. R., Steele A. A., Penegar I. & Campbell S. A. The use of atomic force microscopy for studying interactions of bacterial biofilms with surfaces. Colloids Surf. B Biointerfaces 23, 231–247 (2002).

[b53] Deerinck T. J., Bushong E. A., Thor A. & Ellisman M. H. NCMIR methods for 3D EM: a new protocol for preparation of biological specimens for serial block face scanning electron microscopy. Microscopy 6–8 (2010). Available at http://gatan.actonservice.com/acton/attachment/11413/f-017e/1/-/-/-/-/sbfsem%20sample%20prep%20protocol.pdf?modal=1 (2010) (Accessed:19th April 2016).

[b54] Sugimoto S., Arita-Morioka K., Mizunoe Y., Yamanaka K. & Ogura T. Thioflavin T as a fluorescence probe for monitoring RNA metabolism at molecular and cellular levels. Nucleic Acids Res. 43, e92 (2015). 25883145 10.1093/nar/gkv338 PMC4538803

