# Supplementary material for: Imaging of bacterial multicellular behaviour in biofilms in liquid by atmospheric scanning electron microscopy
Source: Sci Rep. 2016 May 16;6:25889. doi: 10.1038/srep25889 (PMC4867632; doi:10.1038/srep25889)

Supplementary Information

Imaging of bacterial multicellular behaviour in biofilms in liquid by atmospheric scanning electron microscopy

Shinya Sugimoto[1,2,*], Ken-ichi Okuda[1,2], Reina Miyakawa[1], Mari Sato[3], Ken-ichi Arita-Morioka[4,5], Akio Chiba[1], Kunitoshi Yamanaka[4], Teru Ogura[4], Yoshimitsu Mizunoe[1,2], Chikara Sato[3,*]

[1]Department of Bacteriology, The Jikei University School of Medicine, 3-25-8 Nishi-Shimbashi, Minato-ku, Tokyo 105-8461, Japan
[2]Jikei Center for Biofilm Science and Technology, The Jikei University School of Medicine, 3-25-8 Nishi-Shimbashi, Minato-ku, Tokyo 105-8461, Japan
[3]Biomedical Research Institute, National Institute of Advanced Industrial Science and Technology (AIST), Higashi 1-1-1, Tsukuba, Ibaraki 305-8566, Japan
[4]Department of Molecular Cell Biology, Institute of Molecular Embryology and Genetics, Kumamoto University, Chuo-Ku, Kumamoto, 860-0811, Japan
[5]Present address: Advanced Science Research Center, Fukuoka Dental College, 2-15-1 Tamura, Sawara-Ku, Fukuoka, 814-0193, Japan

*Correspondence should be addressed to S.S. (ssugimoto@jikei.ac.jp) and C.S. (ti-sato@aist.go.jp).

Supplementary Methods

Bacterial culture conditions

Escherichia coli strains (Supplementary Table 1) were grown in Luria-Bertani (LB) medium (Merk, Darmstadt, Germany), YESCA medium composed of 1% (w/v) casamino acid (Becton Dickinson, Franklin Lakes, NJ, USA), 0.1% (w/v) yeast extract (Becton Dickinson), and YESCA plate containing 2% (w/v) agar. *Staphylococcus aureus* and *Staphylococcus epidermidis* strains (Supplementary Table 1) were cultured in brain heart infusion (BHI) medium (Becton Dickinson) and BHI supplemented with 1% (w/v) glucose (BHIG) medium, and tryptic soy broth (TSB) medium (Becton Dickinson). If required, appropriate antibiotics were supplemented at the concentration of 100 μg/ml ampicillin, 30 μg/ml chloramphenicol, 50 μg/ml kanamycin, and 50 μg/ml neomycin.

Construction of strains

Mutant strains of *S. aureus* MR23 were constructed using the *E. coli*-*S. aureus* shuttle vector pKOR1[1] according to the procedure modified by Chiba *et al.*[2]. In brief, approximately 500-bp upstream and downstream sequences of the *spa*, *sbi*, and *eap* genes were amplified by PCR from the MR23 genomic DNA using the following primer sets, respectively: *attB1*-*spa*-F and *spa*-R; *spa*-F and *attB2*-*spa*-R; *attB1*-*sbi*-F and *sbi*-R; *sbi*-F and *attB2*-*sbi*-R; *attB1*-*eap*-F and *eap*-R; *eap*-F and *attB2*-*eap*-R (Supplementary Table 2). These fragments were connected by splicing by overlap extension PCR (SOE-PCR)[3]. The created PCR product was cloned into pKOR1 using the Gateway BP Clonase II enzyme mix (Life Technologies, Palo Alto, CA, USA); the resulting plasmids are referred to as pKOR1-Δ*spa*, pKOR1-Δ*sbi*, and pKOR1-Δ*eap* (Supplementary Table 2). Using these plasmids, the *spa*, *sbi*, and *eap* genes were deleted solely or sequentially from the MR23 genomic DNA according to the procedure reported previously[1,2].

We also constructed the MR23 derivative expressing the ClpB::GFP_{uv} translational fusion from the genome. The 500-bp DNA encoding the C-terminus of ClpB, and its downstream 500-bp fragment were amplified from the MR23 genomic DNA by PCR using the following primer sets, respectively: *attB1*-*clpB*-*Cterm*-F and *clpB*-*Cterm*-R; *clpB*-*down*-F and *attB2*-*clpB*-*down*-R. The gfp_{uv} gene was also amplified from pGFP_{uv} (Clontech, Palo Alto, CA, USA) by PCR using the following primer set: *clpB::*gfp_{uv}-F and *clpB::*gfp_{uv}-R (Supplementary Table 2). These fragments were connected and ligated into pKOR1 as described above. The resulting plasmid pKOR1-*clpB::*gfp_{uv} (Supplementary Table 1) was used to generate MR23 expressing the ClpB::GFP_{uv} translational fusion from the genome (Supplementary Table 1).

Oligonucleotide primers (Supplementary Table 2) were synthesized by Life Technologies (Tokyo, Japan).

Enzyme susceptibility of biofilms

To test the susceptibility of filamentous structures in *S. aureus* MR10 biofilms, the biofilms were cultured in BHIG media supplemented with 100 U/ml DNase I (Roche Diagnostics, Mannheim, Germany), 100 μg/ml proteinase K (Sigma, St. Louis, MO, USA), or 20 μg/ml dispersin B (Kane Biotech Inc., Manitoba, Canada) in ASEM dishes for 4 h at 37°C. The biofilms were fixed with 1% glutaraldehyde (GA) for 10 min at room temperature, labelled with positively charged Nanogold (PCG), and imaged by ASEM as described below.

Immuno-labelling for ASEM

Paraformaldehyde (PFA)-fixed staphylococcal biofilms were incubated with 5% skimmed milk and 1% goat serum (Sigma) in buffer A [40 mM HEPES (pH 7.4) and 150 mM NaCl] for 1 h at room temperature. PFA-fixed *E. coli* cells were incubated with 5% skimmed milk (Wako, Tokyo, Japan) in buffer A for 1 h at room temperature.

For primary labelling, the biofilms were incubated with antibodies added to the blocking solution for 1 h at room temperature or overnight at 4°C. Antibodies: rabbit anti-Eap antibody developed by Scrum (Tokyo, Japan, 1/200 dilution in the blocking solution)[2], rabbit anti-curli antibody developed by Scrum (Tokyo, Japan, 1/100 dilution in the blocking solution), rabbit anti-ClpB antibody developed by Eurofins Genomics (Tokyo, Japan, 1/200 dilution in the blocking solution), rabbit anti-DnaK antibody developed by Eurofins Genomics (Tokyo, Japan, 1/200 dilution in the blocking solution), mouse anti-dsDNA antibody (Abcam, Cambridge, MA, USA, 1/200 dilution in the blocking solution), and rabbit anti-Spa antibody (EY Laboratories, San Mateo, CA, 1/500 dilution in Can Get Signal A: TOYOBO, Osaka, Japan). As a control, non-specific purified rabbit IgG (Sigma, 1/200 in the blocking solution) was used instead of the primary antibodies. Fab fragments of rabbit anti-ClpB antibody, rabbit anti-DnaK antibody, and non-specific rabbit IgG were prepared using a Pierce Fab Preparation Kit (Thermo Fisher Scientific, Tokyo, Japan) and were also used for the primary labelling (1/10 dilution in the blocking solution).

For secondary labelling, biofilms were washed 3-times with PBS, incubated with Fab' fragments bound to 1.4-nm Nanogold and Alexa Fluor 594-conjugated goat anti-rabbit IgG (Nanoprobes, 1/2,000-1/4,000 dilution in the blocking solution), with Fab' fragments bound to 10-nm colloidal gold and Alexa Fluor 488-conjugated goat anti-mouse IgG (Nanoprobes, 1/1,000-1/2,000 dilution in the blocking solution), or with 5-nm colloidal gold-conjugated protein A (EY Laboratories, 1/500 in Can Get Signal A) for 1 h at room temperature, and washed at least 3-times with PBS. After post-fixing with 1% GA, the Nanogold particles were enlarged by gold enhancement using GoldEnhance-EM (Nanoprobes) for 10 min at room temperature, followed by washing with DDW. The biofilms were imaged by ASEM. In some cases a counter-staining step was employed as detailed in the figure legends.

ASEM imaging

ASEM images were recorded using the ClairScope ASEM system (JASM-6200, JEOL, Ltd, Tokyo, Japan) (Fig. 1)[4]. The standard 35 mm bio-ASEM dish[4] and the multiple windowed ASEM dish[5] were employed. Staphylococcal biofilms were formed directly in an ASEM dish, while *E. coli* colony biofilms and culture suspensions were placed on an ASEM dish after cultivation. Fixation and staining were performed in the ASEM dishes. The buffer was then exchanged; the biofilms and bacterial cells were immersed in 10 mg/ml D-glucose or ascorbic acid in DDW and observed using the inverted SEM of the ASEM. The acceleration voltage of the SEM was 20 or 30 kV, and backscattered electrons (BSE) from the specimens were recorded by a BSE imaging (BEI) detector to visualise the sample (Fig. 1). The electron dose at the highest magnification of 20,000× was 20 $e^{-}/Å^{2}$, which is less than half the dose permitted in low-dose cryo-electron microscopy aiming at atomic resolution single particle reconstructions.

TEM imaging

S. aureus MR23 biofilm cells were grown in BHIG for 4 h at 37°C in 35-mm plastic dishes (Nunc). Biofilms were scraped, collected by centrifugation, and fixed with 2.5% GA in 0.1 M phosphate buffer

(PB) (pH 7.4) at room temperature for 1 h and further with 1% osmic acid (OA) in PB at 4°C for 1 h. Specimens were dehydrated by treatment with an alcohol gradient series at room temperature, embedded in Epon812, and thin-sectioned using a Leica Ultracut UCT ultramicrotome. Thin sections were stained with uranyl acetate (UA) and lead citrate (LC), and observed with a H7600 TEM (Hitachi, Tokyo, Japan) at 80 kV.

The isolated MV fractions from *S. aureus* biofilm and planktonic cultures were adsorbed to thin carbon films rendered hydrophilic by glow discharge and supported by copper mesh grids, stained with 2 % UA for 10 sec, air-dried and imaged by TEM.

E. coli colony biofilms with or without curli were observed by negative stain TEM as recently reported[6].

Conventional SEM imaging

Biofilms formed on a cover slip were fixed in phosphate buffered saline (PBS) containing 2.5% glutaraldehyde at room temperature for 1 h. After washing with double distilled water three times, the specimens were dehydrated through a series of graded ethanol solutions (50%, 70%, 80%, 90%, 95%, and 99.5%), substituted with t-butyl alcohol, freeze-dried, and coated with platinum. The samples were observed using a Keyence VE-9800 SEM (Keyence, Osaka, Japan) as previously reported[7,8].

Indirect immunofluorescence microscopy

To detect IgG-binding proteins localised on the cell surface, *S. aureus* cells were cultured in BHIG medium in a glass bottom dish for 3 h at 37°C under static conditions. The cells were fixed with 4% PFA and incubated with 4% skimmed milk in PBS with non-specific rabbit IgG (Sigma, 1/1,000 in the blocking solution) for 60 min at room temperature for the primary labelling, and then with Fab' fragments bound to 1.4-nm Nanogold and Alexa Fluor 594-conjugated goat anti-rabbit IgG (Nanoprobes, 1/2,000 dilution in the blocking solution) for 60 min at room temperature for the secondary labelling. In a simple procedure, PFA-fixed cells were incubated with Cy3-conjugated goat anti-rabbit IgG (GE Healthcare, Buckinghamshire, UK, 1/200 dilution in the blocking solution) for 60 min at room temperature. After washing with PBS, the cells were observed using a BZ9000 fluorescence microscope (Keyence).

Sodium dodecyl sulphate-polyacrylamide gel electrophoresis (SDS-PAGE) and Western blotting

The concentrated supernatant of the biofilm culture and the supernatant and pellet fractions obtained by ultracentrifugation as mentioned above, were analysed by SDS-PAGE, followed by Western blotting. The SDS-PAGE gels were used to transfer the separated proteins to a polyvinyl difluoride (PVDF) membrane with a 0.2 μm pore size (ATTO, Tokyo, Japan). The membrane was incubated in blocking buffer composed of 0.3% bovine serum albumin (BSA), 5% goat serum (Sigma), and Tris buffered saline buffer supplemented with 0.1% (w/v) Tween 20 (TBS-T) for 1 h at room temperature. After washing with TBS-T, the membrane was incubated with the primary antibody (rabbit anti-ClpB IgG or rabbit anti-DnaK IgG) 1/5,000 diluted in CanGet Signal 1 (Toyobo) and with the secondary antibody (HRP-conjugated goat anti-rabbit IgG, BioRad, Tokyo, Japan) 1/100,000 diluted in CanGet Signal 2 (Toyobo). After washing with TBS-T, the membrane was treated with ECL Plus (GE Healthcare) and the antibodies signals were recoded using a LAS-4000 imager (GE Healthcare).

Swimming assay

Bacterial swimming activity was examined on a soft agar. An overnight culture of *E. coli* cells grown in LB medium at 30°C was 1/100 diluted into YESCA medium, and incubated for a further 4 h at 30°C. Aliquots of the culture (2 μl) were spotted onto YESCA soft agar plates containing 0.3% agar, and incubated for 24 h at 30°C. The diameter of the colony was recorded.

In parallel, 50 μl of the bacterial culture was placed on the ASEM dish and incubated for 30 min at room temperature. After fixing with 1% GA and 4% PFA, bacteria were labelled with PCG and observed by ASEM to visualise flagella.

Hemolysis assay

Five-microliter aliquots of the MV fraction were spotted on a sheep blood agar plate (Becton Dickinson), and the plate was incubated for 24 h at 37°C. A photo of the plate was then taken using a digital camera.

Coagulase activity assay

Five-microliter aliquots of the MV fraction were spotted on a heat infusion agar plate containing bovine fibrinogen and rabbit serum, and the plate was incubated for 24 h at 37°C. A photo of the plate was then taken using a digital camera.

Supplementary Figure Legends

Supplementary Figure 1. Quantification of *S. aureus* biofilms by a conventional staining method. (**a**) MR23 biofilms formed on 35-mm plastic dishes, stained with 0.2% crystal violet (CV) at the indicated time points and observed. (**b**) MR23 biofilm formation on 96-well polystyrene plates monitored by the absorbance at 595 nm after staining with 0.2% CV. Data points represent the means and standard deviations of results from at least three independent experiments. The standard deviation is less than the size of symbol if no error bars are visible.

Supplementary Figure 2. Air-drying triggers the destruction of intact biofilm structures and the extreme aggregation of biofilm cells. (**a, b**) Schematic diagrams of ASEM observation of biofilms immersed in aqueous solution and under dry condition, respectively. (**c**) *S. aureus* MR23 biofilms stained with osmic acid/uranyl acetate/lead citrate (OA/UA/LC) and observed in 10 mg/ml glucose solution by ASEM. (**d**) The same area as **c** observed by ASEM after the glucose solution had been removed using a pipet and the biofilm allowed to dry. (**e**) *S. aureus* SH1000 biofilms stained with OA/UA/LC observed in 10 mg/ml glucose solution by ASEM. (**f**) The same area as **e** observed by ASEM after removal of the glucose solution and allowing the biofilm to dry. Scale bars, 50 μm.

Supplementary Figure 3. Conventional SEM images of *S. aureus* biofilms formed on glass surfaces. (**a-d**) The proteinaceous biofilm formed by *S. aureus* MR23. (**b**, **c**) Higher magnification images of the white rectangles I and II in **a**. (**d**) Higher magnification image of the white rectangle III in **c**. The cells have rough surfaces. (**e-h**) The polysaccharide-containing biofilm formed by *S. aureus* SH1000. Cells are covered by filamentous and sheet-like matrices. (**g**, **h**) Higher magnification images of the white rectangles

IV and V in **f**. Scale bars, 1 μm.

Supplementary Figure 4. Observation of membrane vesicles by fluorescence microscopy. *S. aureus* MR23 biofilm cells cultured in a glass-bottomed dish were treated with proteinase K. After three washes with PBS, the cells were either treated with Triton X-100 or left untreated, and subsequently labelled with the membrane-binding fluorescent dye FM4-64. Phase contrast, red fluorescence images and merged images are shown. Arrowheads indicate MV-like structures. Scale bars, 10 μm.

Supplementary Figure 5. Visualisation of cytoplasmic proteins encapsulated in MVs secreted from microbes in MR23 biofilm. (**a**) Phase contrast, membranes stained with FM4-64, GFP_{uv}, and merged images are shown. (**b**) Higher magnification merged images showing budding MVs containing GFP_{uv}. Arrowheads indicate GFP_{uv} foci localized close to the cell surface. Scale bars, 10 μm in **a** and 1 μm in **b**.

Supplementary Figure 6. Excretion of cytoplasmic proteins via MV production. Recent studies, including our study[8], reported that cytoplasmic proteins are excreted to the extracellular milieu during biofilm development. Researchers have proposed that cytoplasmic macromolecules, such as proteins, genomic DNA, and ribosomes, are released from bacterial cells via cell lysis or unknown transporting systems. Our ASEM and TEM studies indicate that cytoplasmic proteins, such as molecular chaperone ClpB and GFP_{uv}, might be excreted via an MV-mediated pathway.

Supplementary Figure 7. Virulence factors associated with MVs. Hemolytic activity (**a**) and coagulase (**b**) activities were analysed by spotting the purified MR23 MV fraction (5 μl) onto 5% sheep-blood agar plate and heart infusion agar plate containing bovine fibrinogen and rabbit plasma, respectively. Both hemolytic activity and coagulase activity were observed clearly as circles on the plates. Scale bars, 1 cm.

Supplementary Figure 8. Filamentous structures in *S. epidermidis* biofilms. (**a**) *S. epidermidis* SE4, a clinically isolated strain producing robust polysaccharide-dependent biofilms, was grown in BHIG medium in an ASEM dish at 37°C for 2 h. After removal of the culture medium, bacterial cells were stained with positively charged Nanogold (PCG) and observed as described in Fig. 5. Higher magnification images of the white rectangles I-III in **a** are shown in **b**, **c**, and **d**, respectively. Much higher magnification images of the white rectangles IV in **d** and V in **e** are shown in **e** and **f**, respectively. Arrows indicate filamentous structures. Scale bars, 5 μm in **a-e** and 1 μm in **f**.

Supplementary Figure 9. Lectin-labelling for extracellular polysaccharides. (**a**) *S. aureus* MR10 cells were grown in BHIG medium in a glass-bottomed dish for 2 h at 37°C. The biofilms were fixed with 4% PFA for 10 min at room temperature, labelled with Alexa 488-conjugated wheat germ agglutinin (WGA-Alexa 488), and observed by fluorescence microscopy. (**b**) Higher magnification image of the white rectangles I in **a**. (**c**, **d**) Extracellular matrices (ECM) isolated from *S. aureus* MR10 biofilms by recently developed procedures[2]. The isolated ECMs were untreated (**c**) or treated with dispersin B (**d**), an enzyme hydrolysing staphylococcal polysaccharides. Arrows and arrowheads mark bacterial cells and filamentous extracellular polysaccharides, respectively. Scale bars, 10 μm.

Supplementary Figure 10. Immuno-labelled ASEM without anti-dsDNA mouse IgG as primary antibodies. Four-hour biofilms of MR10 were labelled only with colloidal gold-conjugated anti-mouse IgG secondary antibody. In contrast to Fig. 5d, linearly aligned colloidal gold particles were not observed.

Supplementary Figure 11. PCG-labelling of flagella. Flagella produced by the indicated *E. coli* strains were labelled with PCG and observed in liquid by ASEM. Additional images extending Fig. 6a. Scale bars, 5 μm.

Supplementary Figure 12. Localization of IgG-binding proteins on the surface of *S. aureus* cells. (**a**) Immunofluorescence microscopy images of *S. aureus* MR23 biofilm cells. Phase contrast, DNA probed with DAPI, surface IgG-binding proteins labelled with non-specific rabbit IgG primary antibody and Alexa 594-conjugated secondary antibody, and merged images are shown. Insets: Enlarged images of the white rectangles. (**b**) Schematic diagram of immuno-ASEM for the detection of cell surface IgG-binding proteins, such as Spa and Sbi, using non-specific rabbit IgG primary antibody and FluoroNanogold-conjugated Fab'. (**c**) A typical immuno-ASEM image recorded to detect cell surface IgG-binding proteins; Nanogold particles are visualised as white dots. (**d**) Fluorescence microscopy. Surface IgG-binding proteins were detected using Cy3-labelled non-specific goat IgG in the indicated *S. aureus* MR23 strains. In all images, phase contrast, DAPI, and Cy3 fluorescence images (red) are merged. Double knockout of the *spa* and *sbi* genes drastically reduces non-specific binding of IgG to *S. aureus* cells. (**e**) Biofilms formed by the *S. aureus* MR23 derivatives in 96-well polystyrene plates, were quantified by conventional crystal violet staining; the absorbance at 595 nm was measured. Data points represent the means and standard deviations of results from at least three independent experiments. The deletion of the indicated genes did not affect biofilm development. n.s., not significant. Scale bars, 10 μm in **a** and **d** and 1 μm in **c**.

Supplementary Figure 13. The principle of immuno-labelling and subsequent counter staining for *S. aureus* biofilms. *S. aureus* biofilms were labelled with anti-Eap primary antibody and Nanogold-conjugated secondary antibody. After ASEM observation, the same ASEM dishes were used for further counter staining by the modified NCMIR method.

Supplementary Figure 14. Immuno-labelled ASEM using Fab fragments as primary antibodies. (**a**) Schematic diagram of immuno-labelling for the detection of excreted and cell associated cytoplasmic proteins, such as ClpB and DnaK, in 3-h biofilm of MR23. After immuno-labelling, bacterial cells were further stained with osmic acid and uranyl acetate OA and UA. (**b**) anti-ClpB Fab and (**c**) anti-DnaK Fab fragments were used for primary labelling and Nanogold-conjugated goat anti-rabbit Fab' for secondary labelling. Whole molecules of (**d**) non-specific rabbit IgG and (**e**) its Fab fragment were used as positive and negative controls, respectively. Scale bars, 1 μm.

Supplementary Figure 15. Immuno-ASEM using colloidal gold-conjugated Spa. (**a**) Schematic diagram of immuno-labelling for the detection of cell surface proteins such as a secreted protein: Eap (**b**), a cell wall-anchored protein: Spa (**c**), and excreted and cell associated cytoplasmic proteins: ClpB (**d**) and DnaK (**e**), using the indicated antibodies and colloidal gold-conjugated Spa for primary and secondary labelling, respectively. After the labelling, bacterial cells were further stained with OA and UA. (**f**)

Negative control; the primary antibody was not used, but the secondary antibody, colloidal gold-conjugated Spa, was still added. Scale bars, 1 μm.

Supplementary Figure 16. Curli- and biofilm-producing phenotypes of *E. coli* strains. Curli production of the indicated strains was confirmed by Congo red (CR)-binding assay. Biofilms were formed in 96-well plates, stained with 0.02% CV, and quantified by measuring the absorbance at 595 nm. **, $P<0.01$; n.s., not significant.

Supplementary Movie S1-S6. TEM images of serial thin sections of Epon812 embedded *S. aureus* MR23 biofilm grown for 4 h at 37°C. Images were taken as described in Fig. 4a. Typical images are shown as GIF images.

Supplementary References

1. Bae, T. & Schneewind, O. Allelic replacement in *Staphylococcus aureus* with inducible counter-selection. *Plasmid* **55**, 58-63 (2006).
2. Chiba, A., Sugimoto, S., Sato, F., Hori, S. & Mizunoe, Y. A refined technique for extraction of extracellular matrices from bacterial biofilms and its applicability. *Microb. Biotechnol.* **8**, 392-403 (2015).
3. Ho, S.N., Hunt, H.D., Horton, R.M., Pullen, J.K. & Pease, L.R. Site-directed mutagenesis by overlap extension using the polymerase chain reaction. *Gene* **77**, 51-59 (1989).
4. Nishiyama, H. *et al*. Atmospheric scanning electron microscope observes cells and tissues in open medium through silicon nitride film. *J. Struct. Biol*. **169**, 438-449 (2010).
5. Memtily, N. *et al*. Observation of tissues in open aqueous solution by atmospheric scanning electron microscopy: applicability to intraoperative cancer diagnosis. *Int. J. Oncol.* **46**, 1872-1882 (2015).
6. Arita-Morioka, K., Yamanaka, K., Mizunoe, Y., Ogura, T. & Sugimoto, S. Novel strategy for biofilm inhibition by using small molecules targeting molecular chaperone DnaK. *Antimicrob. Agents Chemother.* **59**, 633-641 (2015).
7. Sugimoto, S. *et al*. AAA+ chaperone ClpX regulates dynamics of prokaryotic cytoskeletal protein FtsZ. *J. Biol. Chem.* **285**, 6648-6657 (2010).
8. Sugimoto, S. *et al*. *Staphylococcus epidermidis* Esp degrades specific proteins associated with *Staphylococcus aureus* biofilm formation and host-pathogen interaction. *J. Bacteriol.* **195**, 1645-1655 (2013).
9. Baba, T. *et al*. Construction of *Escherichia coli* K-12 in-frame, single-gene knockout mutants: the Keio collection. *Mol. Syst. Biol.* **2**, 2006.0008 (2006).
10. Horsburgh, M.J. *et al. sigmaB* modulates virulence determinant expression and stress resistance: characterization of a functional *rsbU* strain derived from *Staphylococcus aureus* 8325-4. *J. Bacteriol.* **184**, 5457-5467 (2002)

Supplementary Table 1. Strains and plasmids used in this study

Strain or plasmid	Description[a]	Source or reference
E. coli strain		
BW25113	Δ*(araD-araB)567*, Δ*lacZ4787(::rrnB-3)*, λ^-, *rph-1*, Δ*(rhaD-rhaB)568*, *hsdR514* K-12 wild type strain (Keio collection)	9
JW1020	BW25113 Δ*csgG*::Km^R	9
JW1023	BW25113 Δ*csgD*::Km^R	9
JW1025	BW25113 Δ*csgA*::Km^R	9
JW1908	BW25113 Δ*fliC*::Km^R	9
JW4277	BW25113 Δ*fimA*::Km^R	9
S. aureus strain		
SH1000	*S. aureus* strain 8325-4 with functional *rsbU*, PIA/eDNA-dependent biofilm producer	10
MR10	MRSA strain isolated in the Jikei Hospital, PIA/eDNA-dependent biofilm producer	2, 8
MR23	MRSA strain isolated in the Jikei Hospital, protein/eDNA-dependent biofilm producer	2, 8
MR23 Δ*spa*	The *spa* gene deleted in MR23	This study
MR23 Δ*spa* Δ*sbi*	The *spa* and *sbi* genes deleted in MR23	This study
MR23 Δ*spa* Δ*sbi* Δ*eap*	The *spa*, *sbi*, and *eap* genes deleted in MR23	This study
MR23 *clpB::*gfp_{uv}	The *clpB* gene replaced to *clpB::*gfp_{uv} in MR23	This study
MR23 GFPuv	MR23 transformed with $pP1GFP_{uv}$	This study
S. epidermidis strain		
SE4	*S. epidermidis* strain isolated in the Jikei Hospital, PIA/eDNA-dependent biofilm producer	2
Plasmid		
$pGFP_{uv}$	gfp_{uv}, pUC replicon, *lac* promoter; Ap^R	Clontech
pKOR1	An *E. coli*/*S. aureus* shuttle vector for knockout of staphylooccal genes by allelic exchange, Cm^R, Ap^R	1
pKOR1-Δ*spa*	A pKOR1-derivative plasmid for knockout of the MR23 *spa* gene, Cm^R, Ap^R	This study
pKOR1-Δ*sbi*	A pKOR1-derivative plasmid for knockout of the MR23 *sbi* gene, Cm^R, Ap^R	This study
pKOR1-Δ*eap*	A pKOR1-derivative plasmid for knockout of the MR23 *eap* gene, Cm^R, Ap^R	This study
pKOR1-*clpB::*gfp_{uv}	A pKOR1-derivative plasmid for replacement of the MR23 *clpB* gene to the *clpB::*gfp_{uv} gene, Cm^R, Ap^R	This study
$pP1GFP_{uv}$	The *sarA* promoter P1 fragment of *S. aureus* SH1000 was ligated with gfp_{uv} gene by SOEing PCR and the amplified fragment was cloned in pNCMO2 using *Pst*I and *Hin*dIII sites; Ap^R Nm^R	2, 8

[a] Ap^R, ampicillin-resistance; Cm^R: Chloramphenicol-resistance; Km^R, kanamycin-resistance; Nm^R, neomycin-resistance.

Supplementary Table 2. Oligonucleotide primers used in this study

Name	Sequence (5' to 3')[a]
attB1-spa-F	ggggacaagtttgtacaaaaaagcaggctgaacgctcaactgaagatgaaag
spa-R	gatatctatcgttgtgtattgtttgtttaatataacgaattatgtattgcaatac
spa-F	gtattgcaatacataattcgttatattaaacaaacaatacacaacgatagatatc
attB2-spa-R	ggggaccactttgtacaagaaagctgggtcattacttgtggcagctaacac
attB1-sbi-F	ggggacaagtttgtacaaaaaagcaggctactctccctgtttgttaactttcg
sbi-R	aaactagagaagatagtgtattccctttctttttacatattaaatttatt
sbi-F	agaaagggaatacactatcttctctagttttacatcatttttttaaataat
attB2-sbi-R	ggggaccactttgtacaagaaagctgggttgcaccttggccgatatcttc
attB1-eap-F	ggggacaagtttgtacaaaaaagcaggctataaaagttcatgatgttgaccc
eap-R	gaattacacaaaaaaggagagataattttctttaattttataataaggcatctcac
eap-F	gtgagatgccttattataaaattaaagaaaattatctctcctttttgtgtaattc
attB2-eap-R	ggggaccactttgtacaagaaagctgggttcagtaagtgcaccaactaataataaat
attB1-clpB-Cterm-F	ggggacaagtttgtacaaaaaagcaggcttgaggttgaaaaagcgcatactgacg
clpB-Cterm-R	gttcttctcctttactcataccaccaccaccaccttcatgaatttttcaacattaaacgttaagttattgtctg
clpB-down-F	cacatggcatggatgagctctacaaataaaattgaaccaagaatgtgatgattaatcgc
attB2-clpB-down-R	ggggaccactttgtacaagaaagctgggtcaacctaaactaagagaagttcattctg
clpB::gfp$_{uv}$-F	tgttgaaaaaattcatgaaggtggtggtggtggtatgagtaaaggagaagaacttttcactggag
clpB::gfp$_{uv}$-R	gcgattaatcatcacattcttggttcaattttatttgtagagctcatccatgccatgtg

[a] Underlines indicate the *attB1* or *attB2* sequence.

Sugimoto *et al*. Supplementary Figure 1

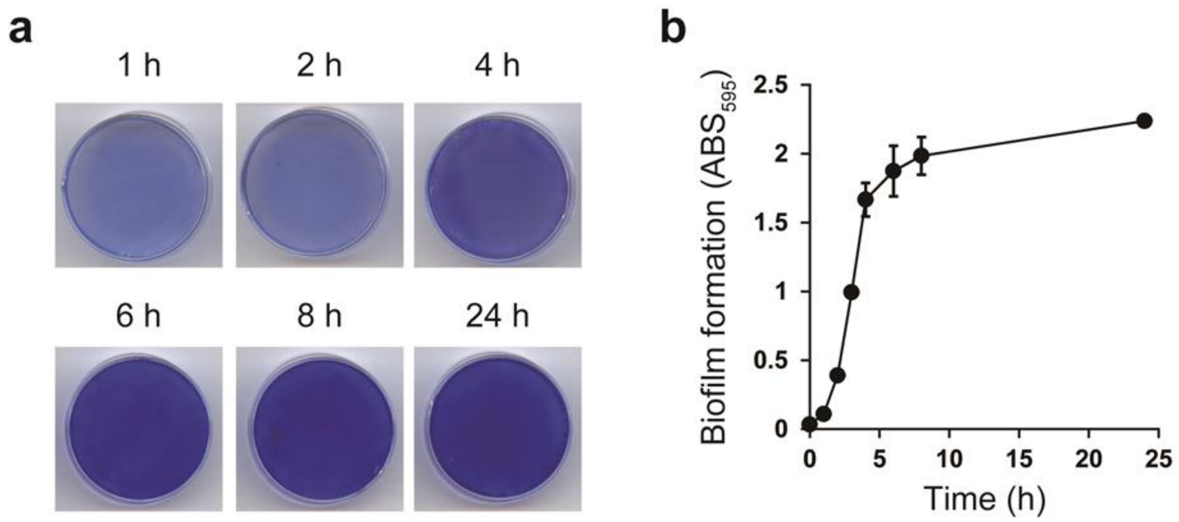

Sugimoto *et al*. Supplementary Figure 2

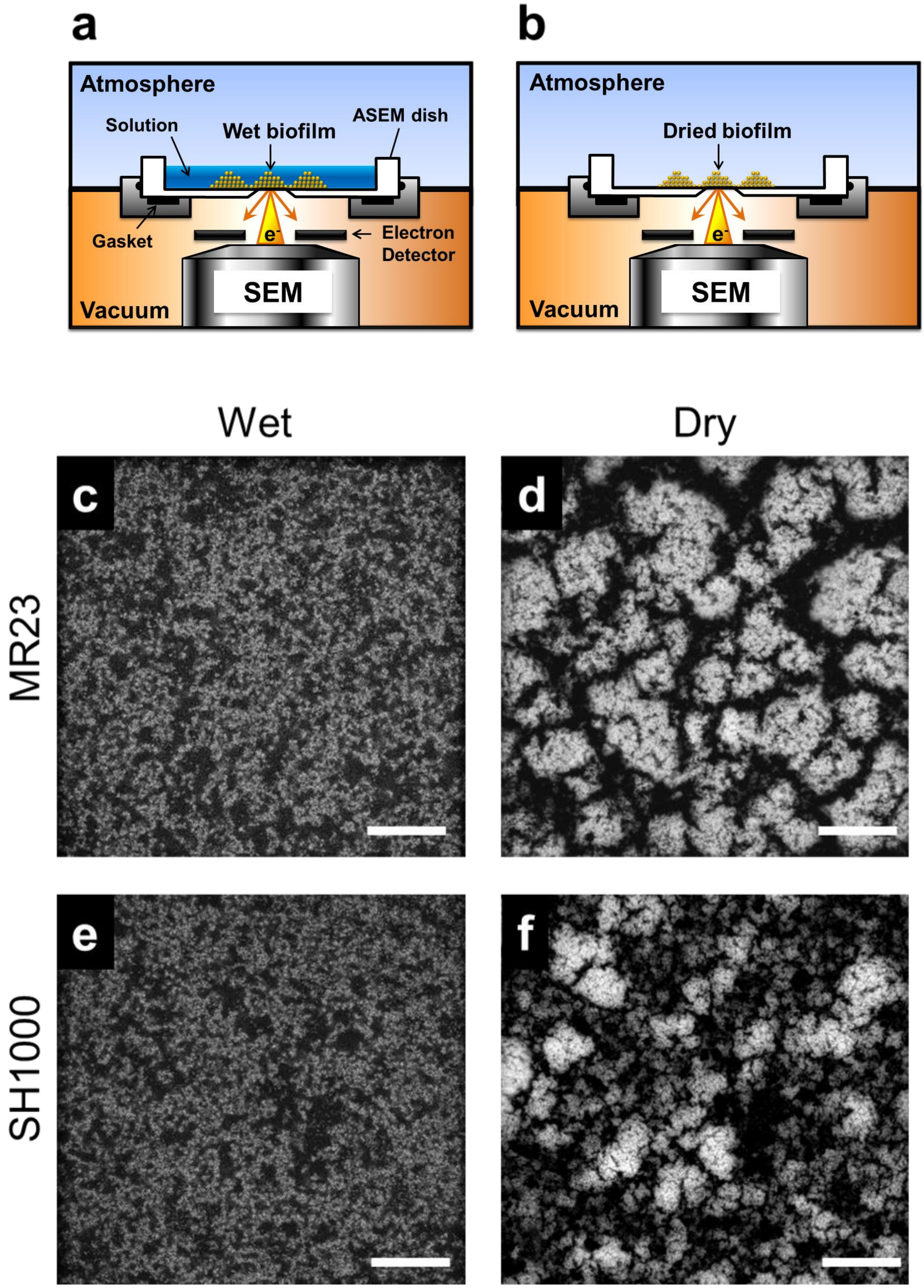

Sugimoto *et al*. Supplementary Figure 3

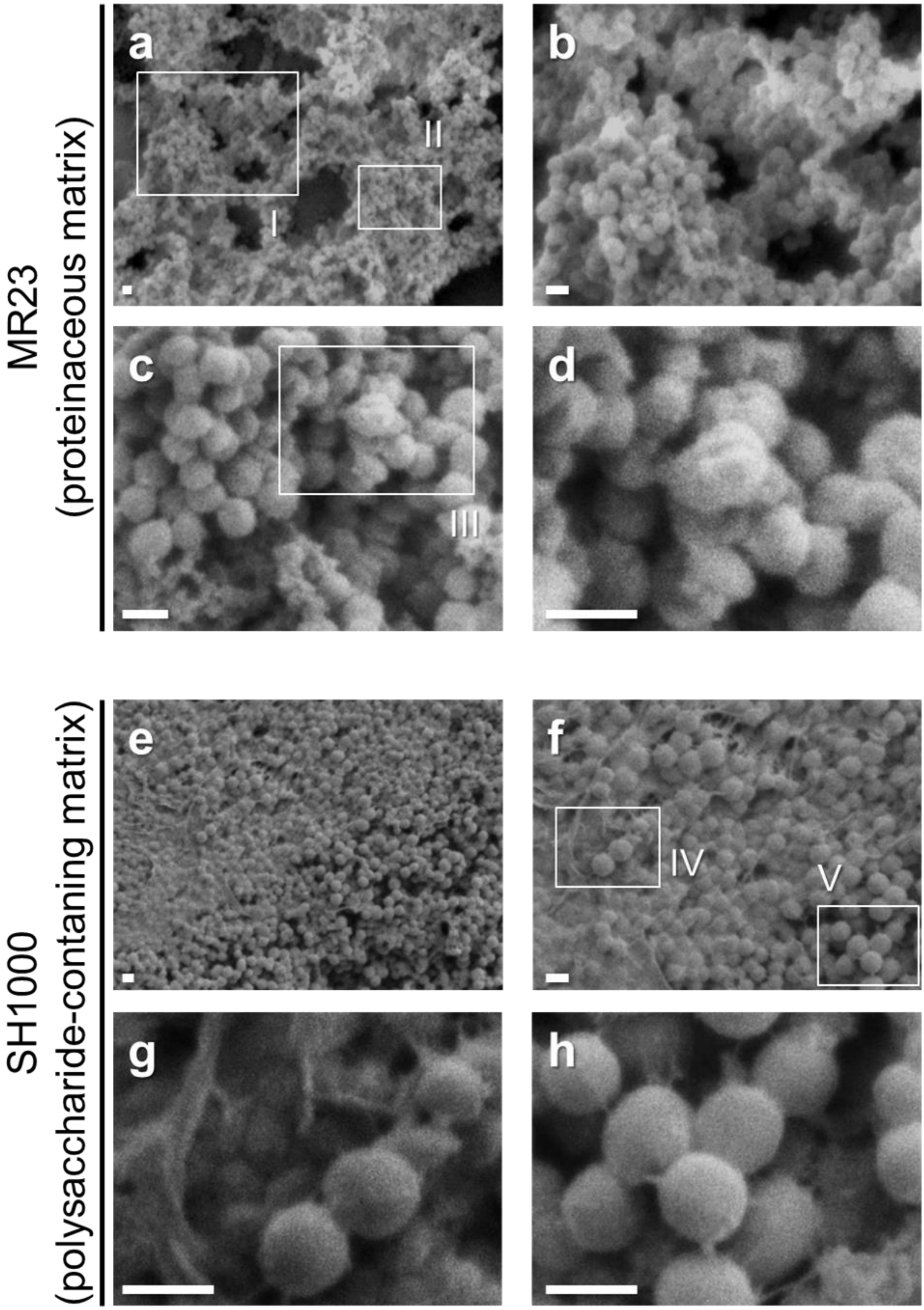

Sugimoto *et al*. Supplementary Figure 4

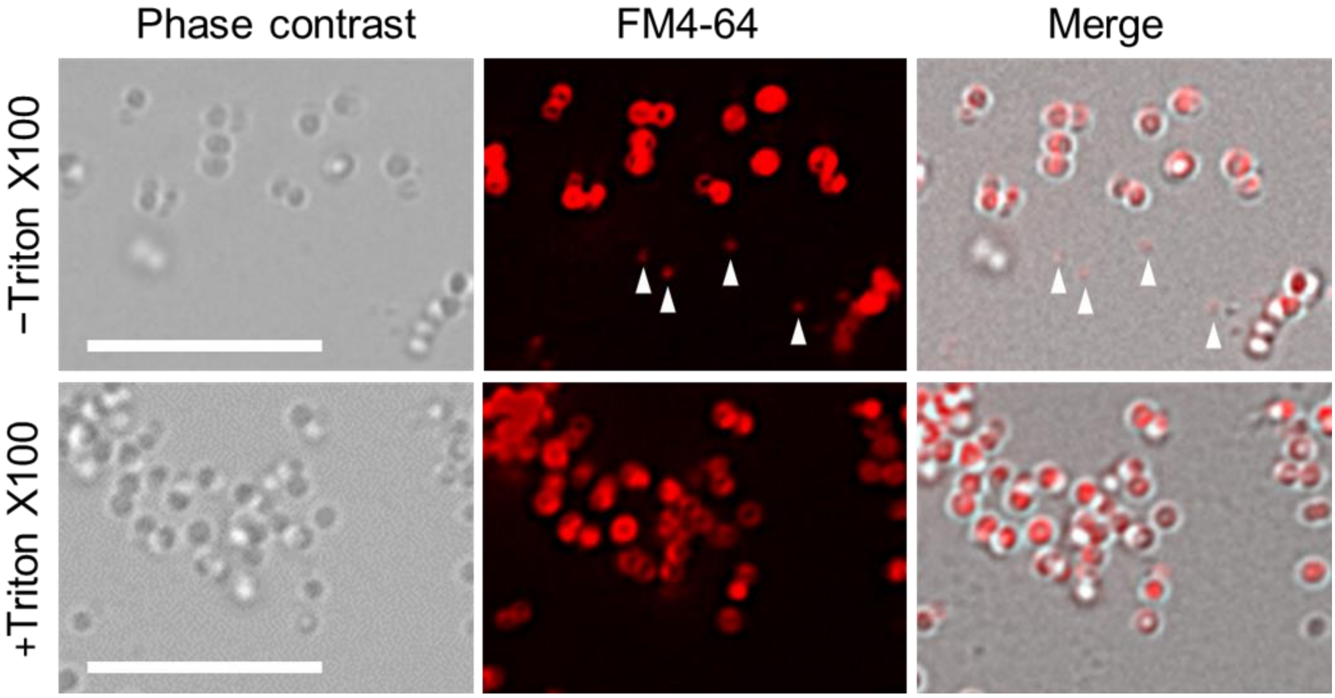

Sugimoto *et al*. Supplementary Figure 5

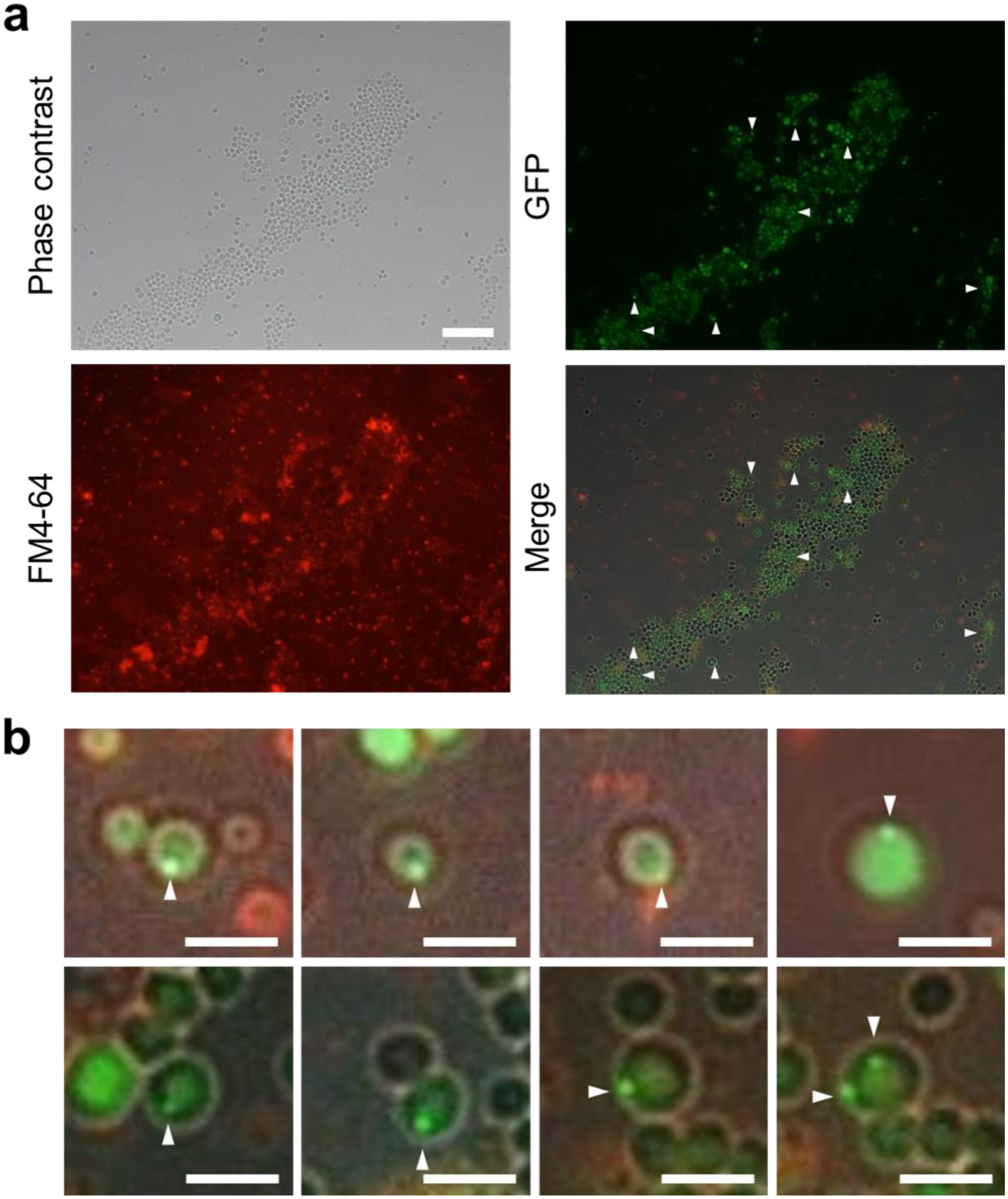

Sugimoto *et al*. Supplementary Figure 6

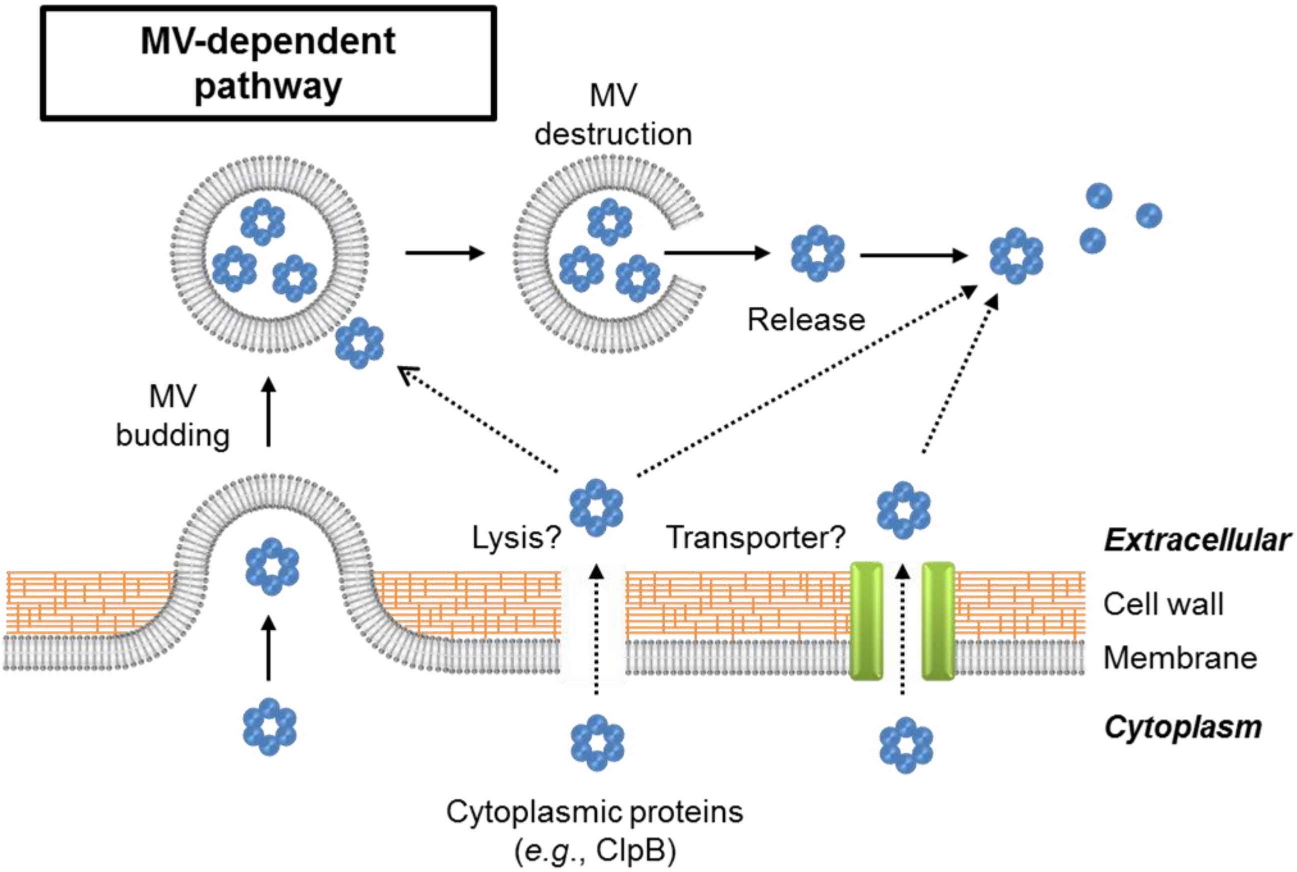

Sugimoto *et al*. Supplementary Figure 7

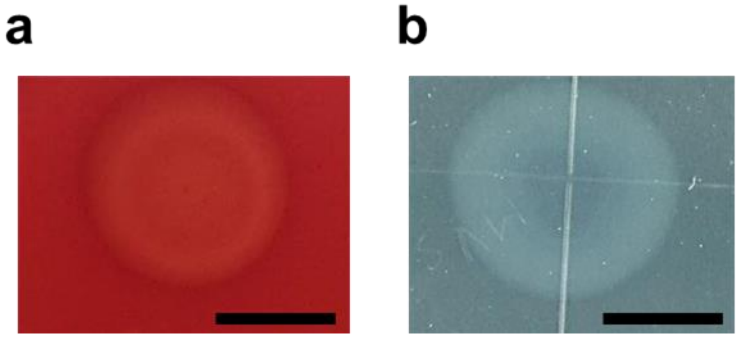

Sugimoto *et al*. Supplementary Figure 8

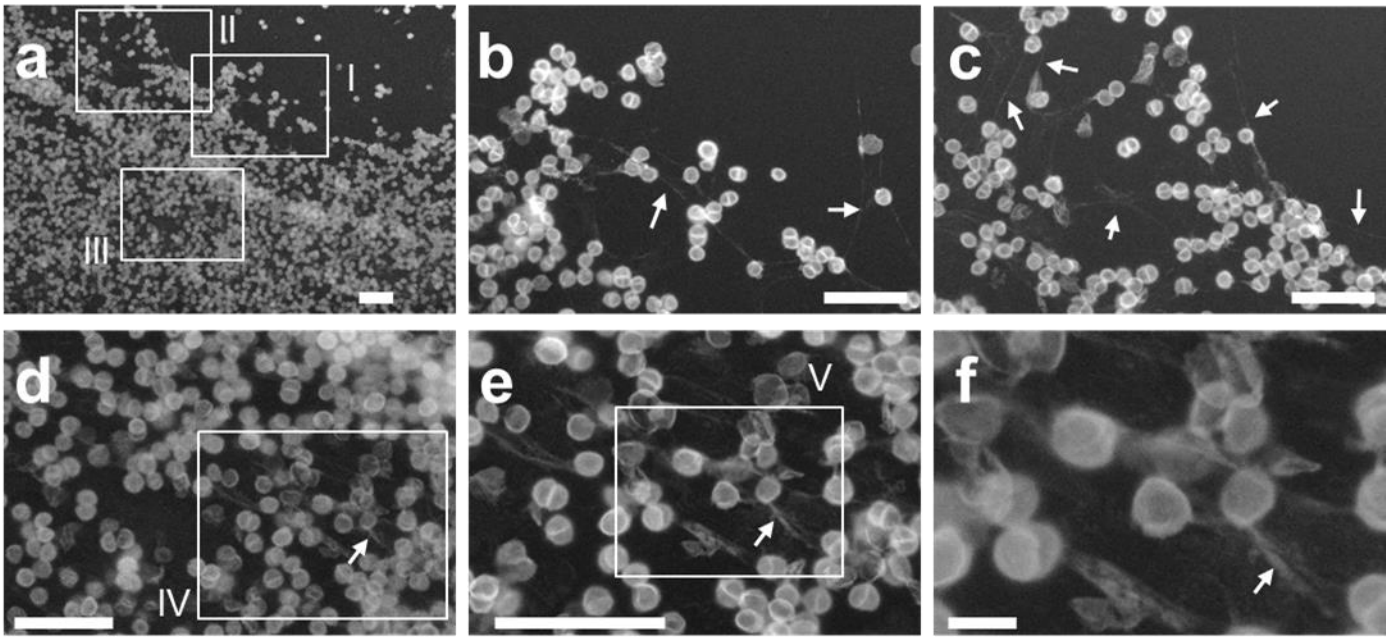

Sugimoto *et al*. Supplementary Figure 9

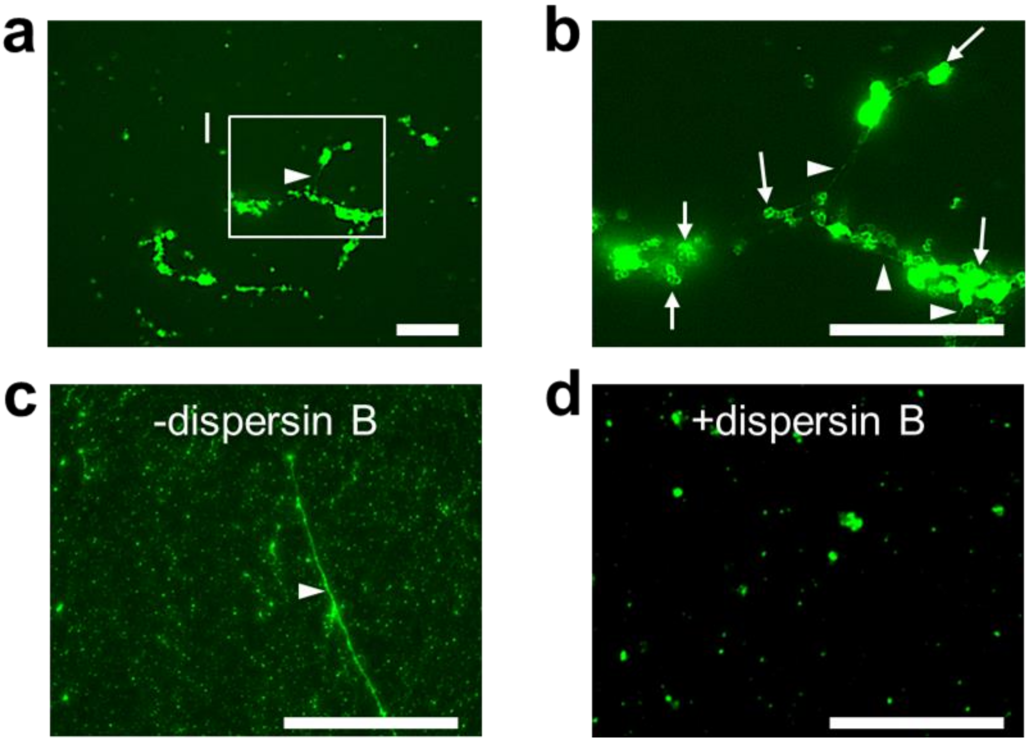

Sugimoto *et al*. Supplementary Figure 10

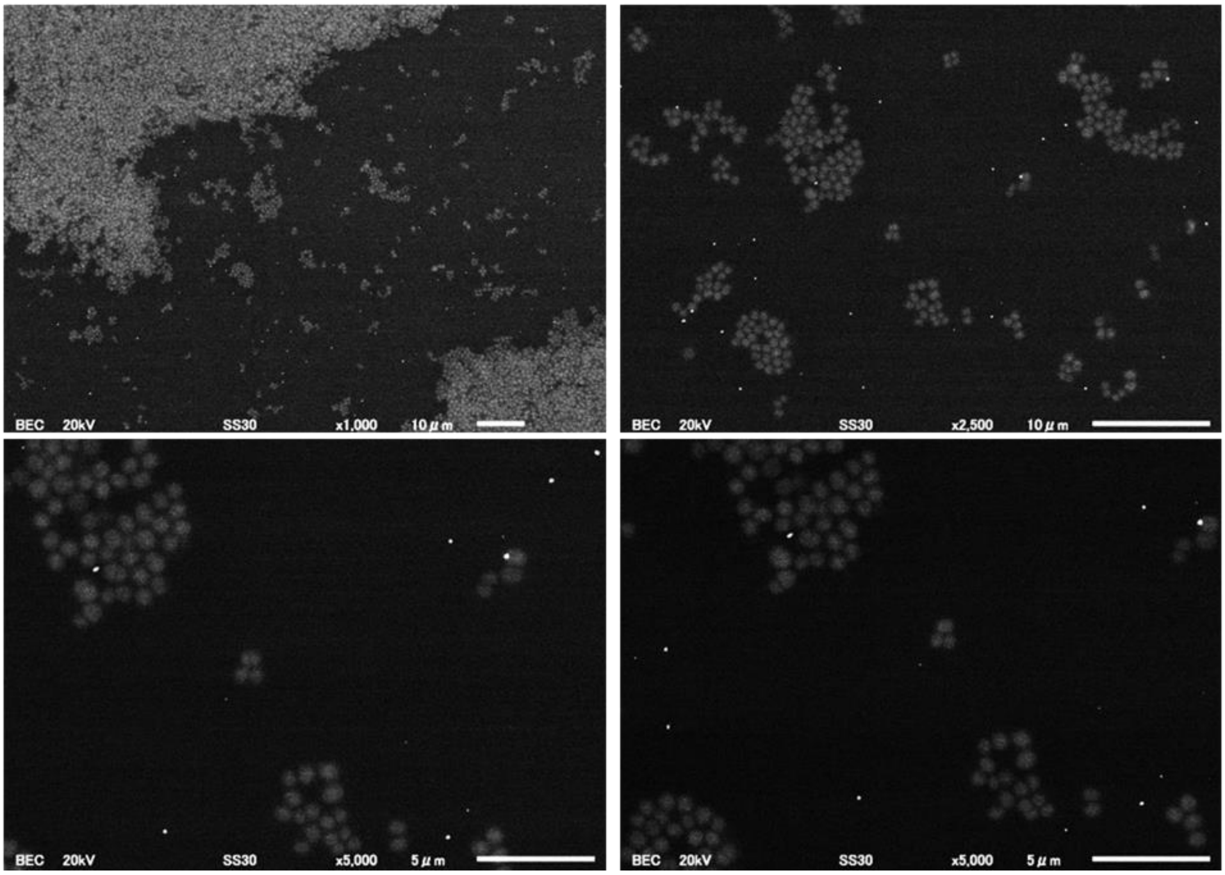

Sugimoto *et al*. Supplementary Figure 11

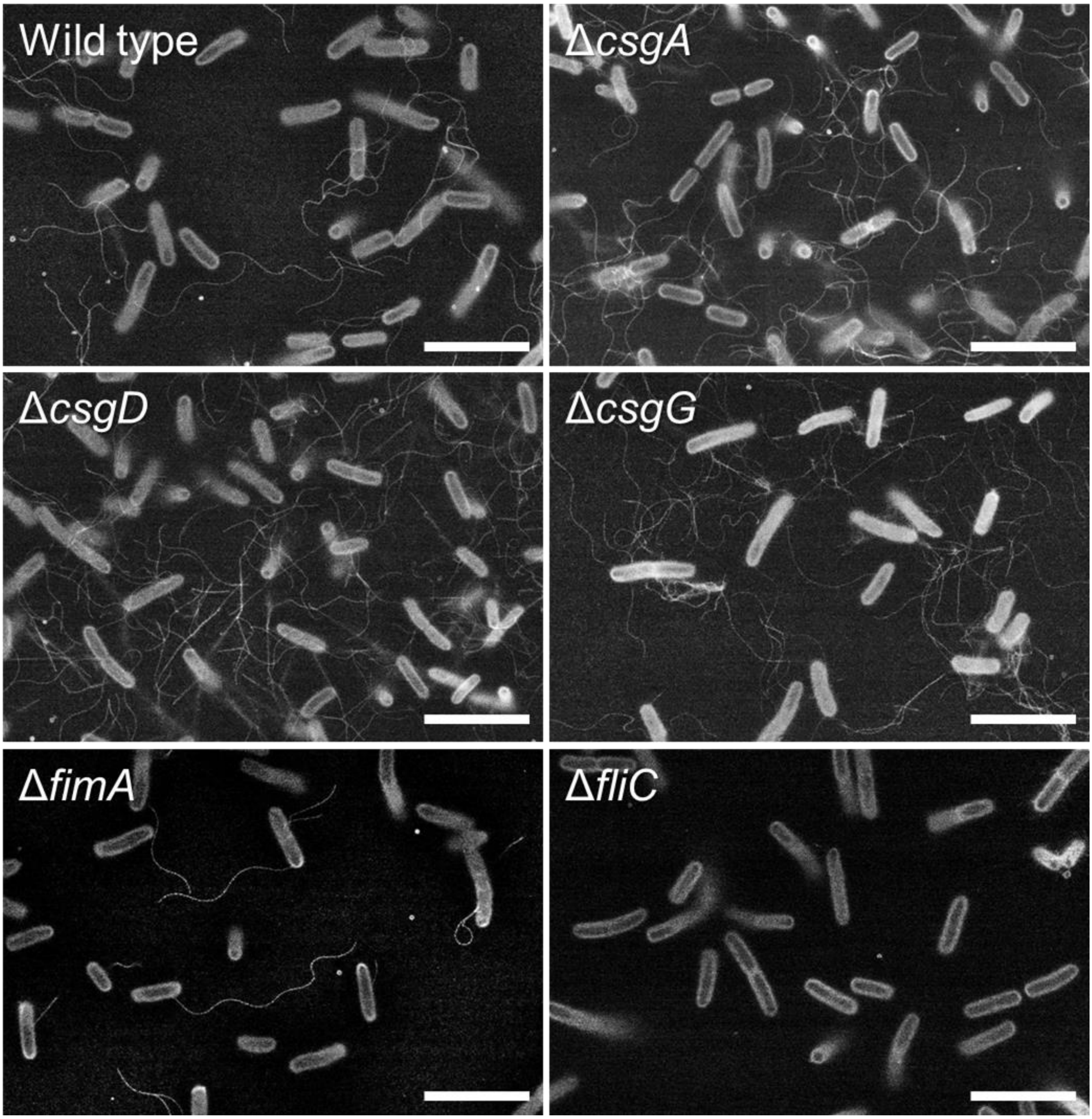

Sugimoto *et al*. Supplementary Figure 12

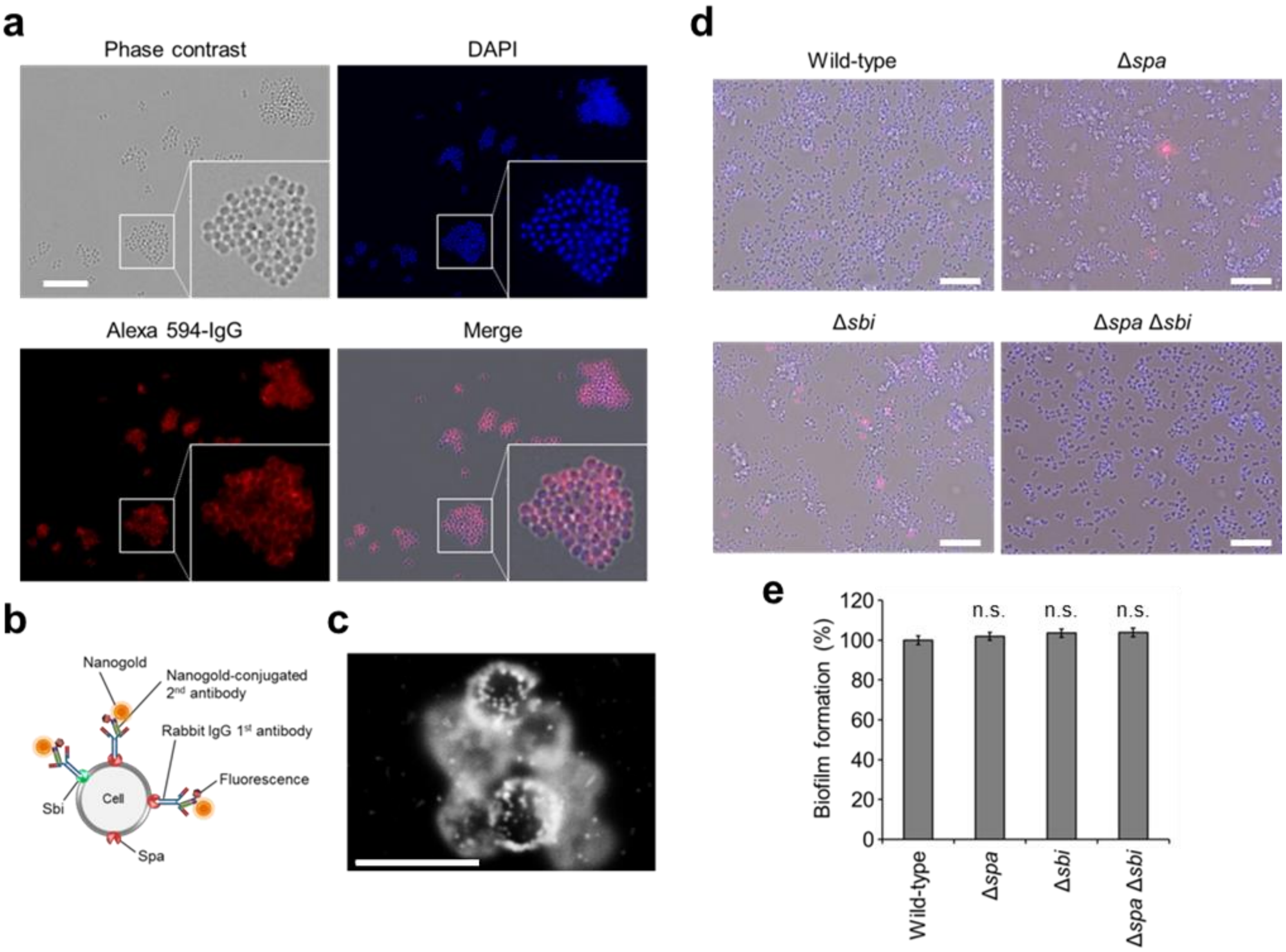

Sugimoto *et al*. Supplementary Figure 13

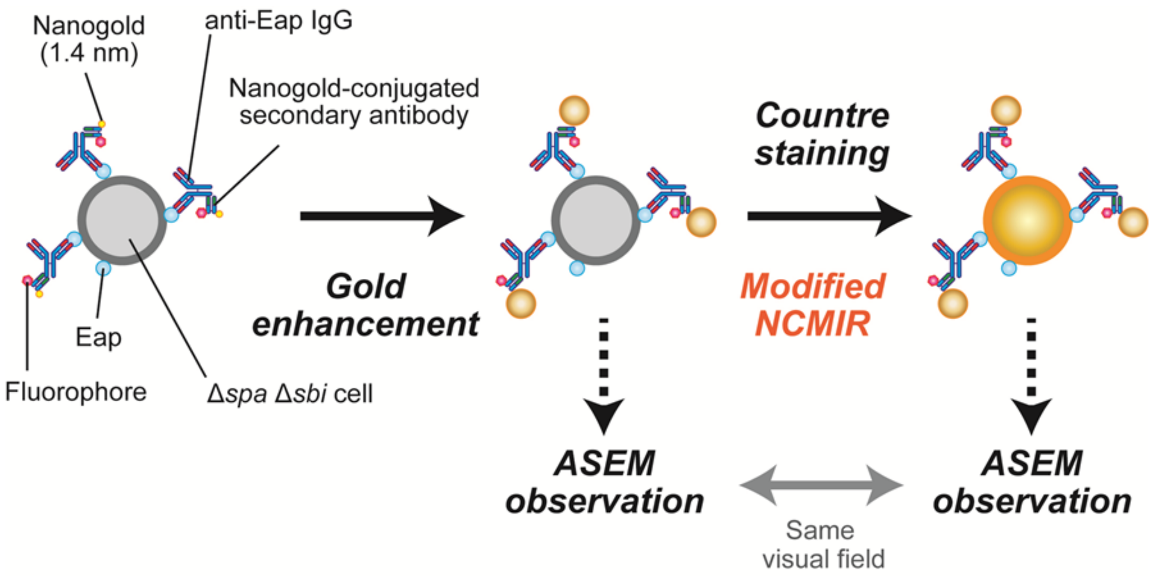

Sugimoto *et al*. Supplementary Figure 14

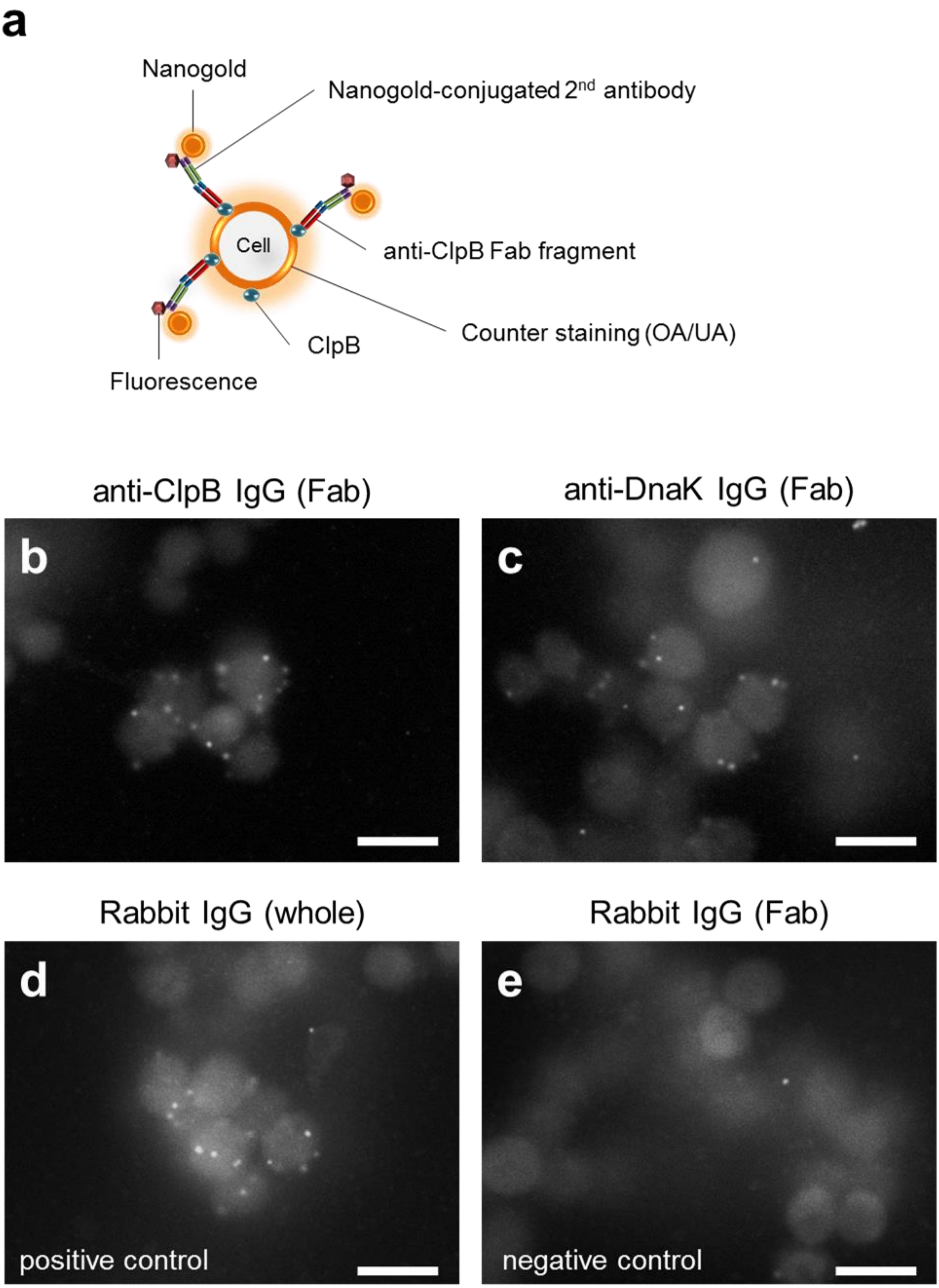

Sugimoto *et al*. Supplementary Figure 15

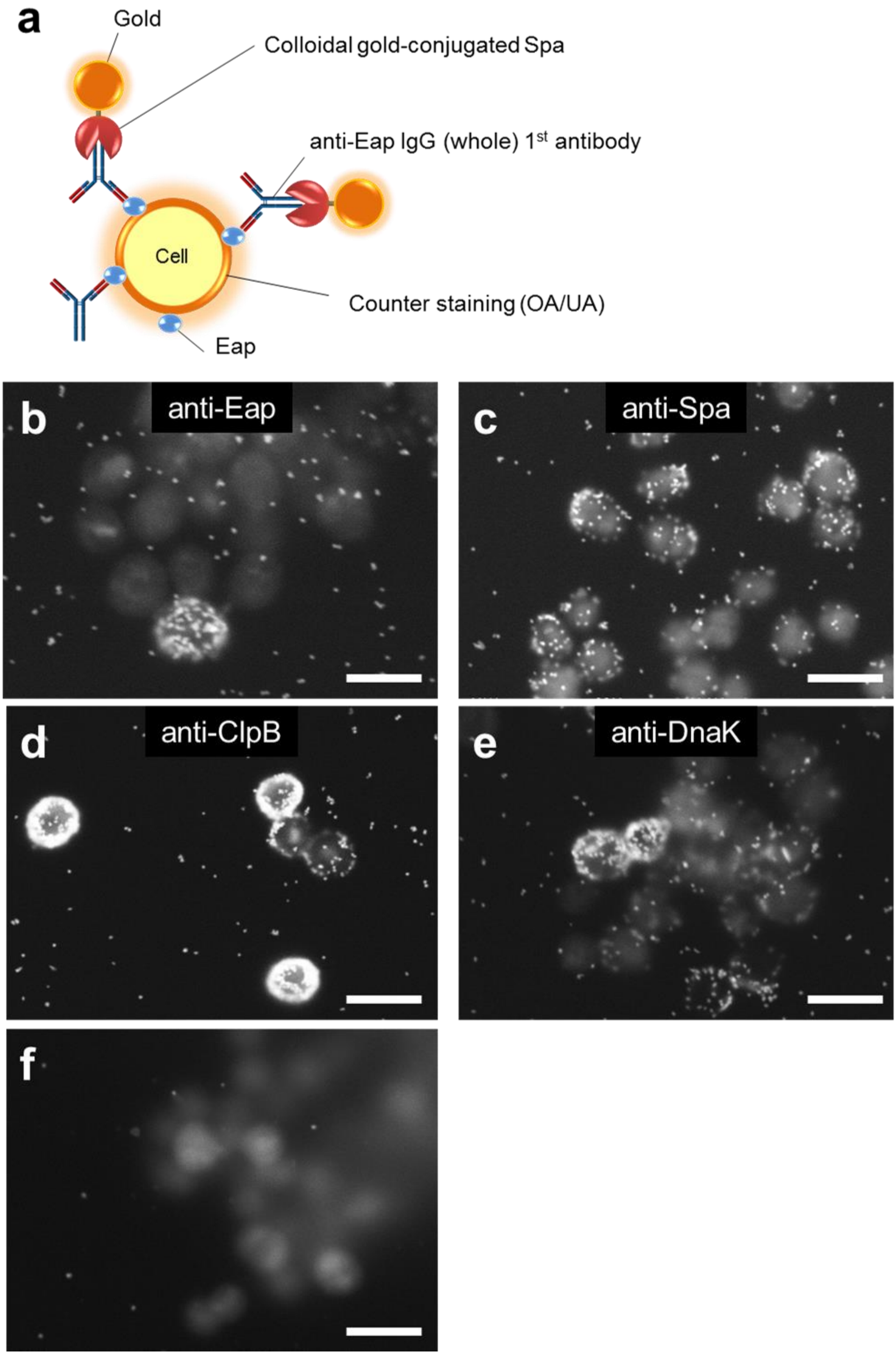

Sugimoto *et al*. Supplementary Figure 16

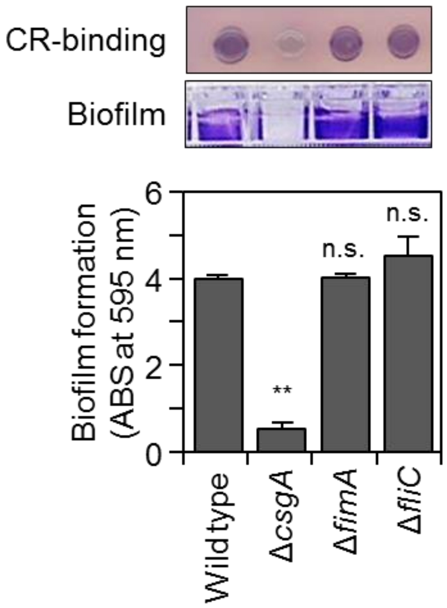

Supplement: Supplementary Information [file srep25889-s1.pdf]
